# Prognostic Role of Ammonia in Critical Care Patients Without Known Hepatic Disease

**DOI:** 10.3389/fmed.2020.589825

**Published:** 2020-10-22

**Authors:** Lina Zhao, Joseph Harold Walline, Yanxia Gao, Xin Lu, Shiyuan Yu, Zengzheng Ge, Huadong Zhu, Yi Li

**Affiliations:** ^1^Emergency Department, Peking Union Medical College Hospital, Peking Union Medical College, Chinese Academy of Medical Sciences, Beijing, China; ^2^Accident and Emergency Medicine Academic Unit, Prince of Wales Hospital, The Chinese University of Hong Kong, Hong Kong, China; ^3^Emergency Department, The First Affiliated Hospital of Zhengzhou University, Zhengzhou, China

**Keywords:** hyperammonemia, non-hepatic, risk factor, prognosis prediction, ammonia

## Abstract

**Background and Aims:** Hyperammonemia usually develops because of hepatic disease, but it may occur in patients with non-hepatic hyperammonemia (NHH). But, studies on the prognosis and possible risk factors of this disorder are lacking. The aim of this study was to find possible prognostic and risk factors for NHH in critically ill patients.

**Methods:** Data were extracted from MIMIC III Database. Survival was analyzed by the Kaplan-Meier method. Univariate and multivariate analyses were performed to identify prognostic factors.

**Results:** Valproic acid, carbamazepine, corticosteroids, recent orthopedic surgery, epilepsy, disorders of urea cycle metabolism, and obesity were found to be risk factors for NHH. Patients in the hyperammonemia group had a higher 30 day mortality than those in the non-hyperammonemia group. After final regression analysis, ammonia was found to be independent predictors of mortality.

**Conclusion:** Ammonia was an independent prognostic predictor of 30 day mortality for critical care patients without liver disease.

## Introduction

Ammonia is the main metabolite of amino acids. Elevated blood ammonia levels can cause encephalopathy (1). Hyperammonemia is most common in patients with acute liver failure or chronic liver disease, but can occur in patients without liver problems (2–4). Elevated blood ammonia levels without a history of liver disease is defined as non-hepatic hyperammonemia (NHH). NHH can be quite common in critically ill patients (up to 73% in a recent study) (5). NHH can occur in patients with a variety of serious conditions, such as intracranial hypertension (6) or congestive heart failure, malnutrition, infectious enterocolitis, or lung transplantation (7–11). Patients with severe heart disease and low serumammonia levels have a significantly lower mortality than patients with persistently high ammonia levels (12). In addition, the inflammatory response and multiple organ dysfunction in patients with sepsis are aggravated by higher blood ammonia levels (13, 14). NHH prolongs intensive care unit (ICU) stays and has been associated with higher mortality (6).

Past studies have mostly focused on hyperammonemia caused by liver disease. Clinically, hyperammonemia caused by non-liver disease may be ignored or misdiagnosed. There have been a few small sample size studies and case reports of NHH (15). NHH has been associated with organ failure, prolonged fasting, and urinary tract infections (16), but studies investigating the prognostic or risk factors on this topic are scarce. The objective of this retrospective cohort study was to determine what risk factors were associated with the development of NHH after hospital admission in critical care patients.

## Materials and Methods

### Database

Data from the MIMIC (Medical Information Mart for Intensive Care) Critical Care Database were used for conducting this study (17). Patients admitted to the ICU of Beth Israel Deaconess Medical Center from 2001 to 2012 were enrolled (17). The project was approved by the institutional review boards of the Massachusetts Institute of Technology and Beth Israel Deaconess Medical Center; there was no requirement for individual patient consent because the project did not impact clinical care and all protected health information was deidentified. The raw data were extracted using structure query language (SQL) with Navicat and further processed with R software. A blood ammonia level >35 μmol/L was defined as hyperammonemia in the MIMIC -III Database. The MIMIC III database (version 1.4) is publically available from https://mimic.physionet.org/.

The establishment of the database was approved by the Massachusetts Institute of Technology (Cambridge, MA) and the Institutional Review Boards of Beth Israel Deaconess Medical Center (Boston, MA). This work has been performed according to the Code of Ethics of the World Medical Association (Declaration of Helsinki).

### Patient Population

Inclusion criteria were as follows: patients (1) ≥18 and ≤89 years-old, (2) admission time >24 h in the ICU, and (3) record contains blood ammonia levels.

Exclusion criteria: (1) patients with acute and chronic liver diseases, including: hepatitis, hepatic cirrhosis, hepatic encephalopathy, hepatorenal syndrome, hepatic injury, or other chronic liver disease were excluded using International Classification of Diseases, Ninth Revision (ICD-9) diagnosis codes on patient discharge (see Supplementary Material), (2) patients having no vital signs or ICD 9 diagnostic code(s) were also excluded.

### Data Extraction and Management

R statistical software (R Foundation for Statistical Computing, Vienna, Austria) was used to retrieve patient information from the MIMIC III Database. The following basic patient data were collected from each patient: age, sex, heart rate (HR), systolic blood pressure (SBP), diastolic blood pressure (DBP), respiratory rate (RR), and temperature (T). The following biochemical test results were also collected from each patient: alanine aminotransferase (ALT), aspartate aminotransferase (AST), partial thromboplastin time (PTT), international normalized ratio (INR), prothrombin time (PT), white blood cell count (WBC), hemoglobin, platelet, blood urea nitrogen (BUN), creatinine (Cr), and glucose. Patients' simplified acute physiology score (SAPSII), quick sequential organ failure assessment (qSOFA) score, sequential organ failure assessment (SOFA), Glasgow coma scale (GCS) were also recorded. Patients' first care unit (i.e., the type of ICU in which they were treated) was recorded based on the data obtained during the first 24 h of each patient's ICU's stay: intensive care unit (ICU). The maximum and minimum values of sodium and potassium were retrieved during the first 24 h of each patient's ICU's stay. The maximum value of ammonia was retrieved during each patient's ICU stay, and the worst scores and laboratory parameters as well as the mean value of vital signs during the first 24 h of ICU admission were used in this study.

### Statistical Analysis

Data distribution was tested using the Shapiro-Wilk test. Patient characteristics were described using median (P25, P75) (interquartile range [IQR]), or frequency and percentage, as appropriate. A non-parametric test (Mann-Whitney U test or Kruskal-Wallis test) was applied for data with non-normal distribution or heterogeneity of variances. Categorical data were compared using the Pearson Chi-squared test, Kaplan-Meier curves were analyzed using log-rank tests. The Cox regression model was used to analyse the independent effects of various parameters on 30 day mortality. Statistical significance was defined as *p* < 0.05. All statistical analyses were performed with R software (version 3.4.3).

## Results

### Baseline Patient Characteristics

**Figure 1:**
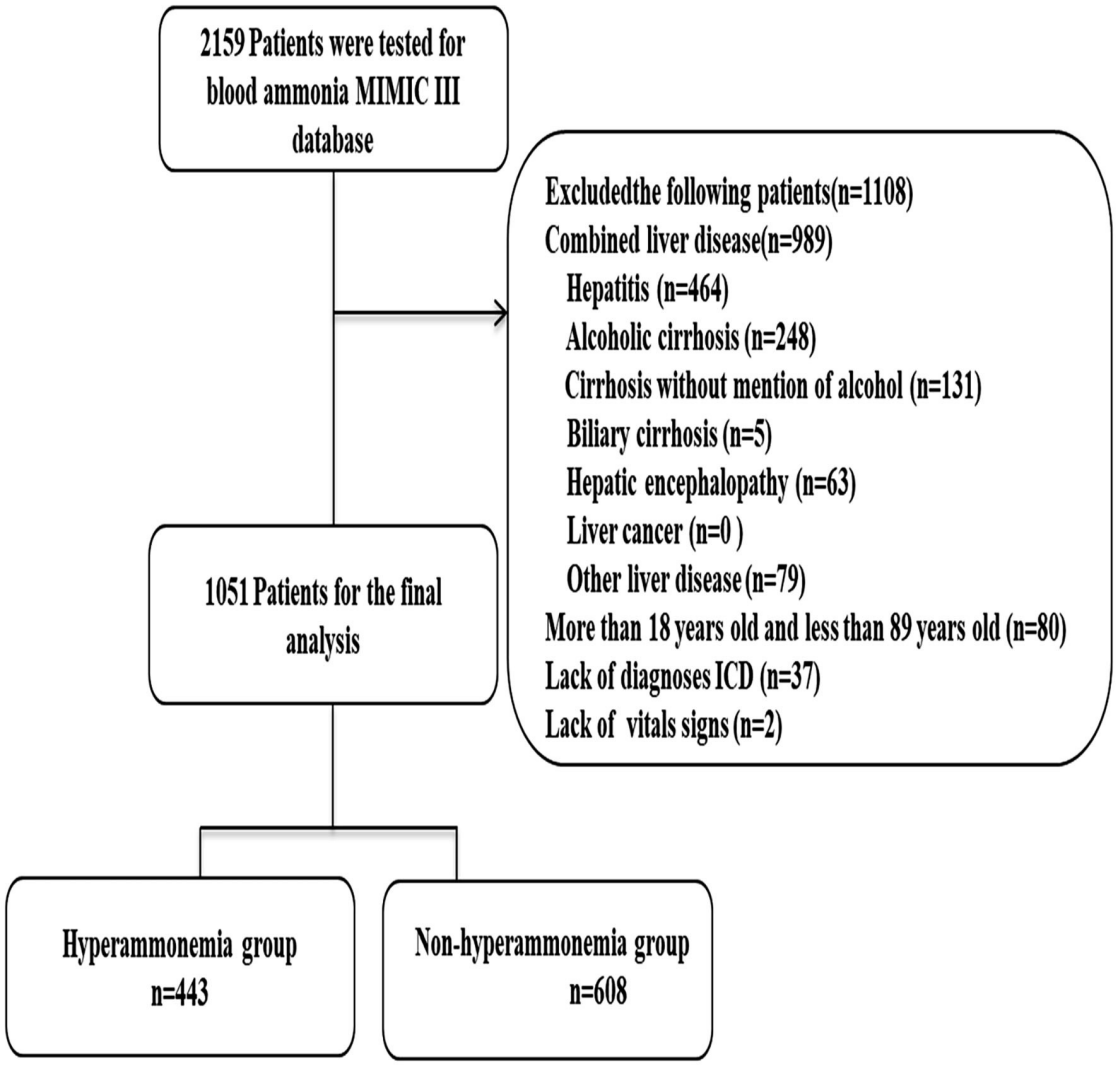
Flow chart of patient selection. MIMIC-III, Medical Information Mart for Intensive Care III.

A total of 1,051 patients were included in this study. Patients were divided into either a hyperammonemia group (*n* = 443) or a non-hyperammonemia group (*n* = 608). Variables with missing data are relatively common in the MIMIC III database. The percentage of missing values for lactate (33.2%), albumin (45.6%), bilirubin (71.3%), pH (25.7%), SpO2 (27.2%) were significant, which were excluded from this study. The percentage of missing values of PTT (11.23%), INR (10.6%), PT (10.6%), ALT (12.3), AST (12.3) were <13%, and the other variables included were <2%. We replaced any missing values of the included variables with multiple imputation. The detailed process of data extraction is shown in Figure 1.

The baseline characteristics, vital signs, laboratory parameters, diagnoses, microbiology results, drug types used, surgeries performed and outcomes for the patients are summarized in Table 1. Differences in age, sex, systolic blood pressure, INR, PT, and ammonia between the hyperammonemia group and the non-hyperammonemia group were statistically significant. The incidence of obesity (8.8 vs. 4.8%) and orthopedic surgery (4.5 vs. 2.5%), corticosteroids (61.6 vs. 44.4%), carbamazepine (8.1 vs. 3.1%), valproic acid (9.7 vs. 5.9%), epilepsy (19.0 vs. 10.9%), and disorders of urea cycle metabolism (0.9 vs. 0.0%) were significantly higher in patients with hyperammonemia than in patients with non-hyperammonemia. In our cohort, ammonia levels showed no correlation with sepsis, gastrointestinal bleeding, intestinal infections, urinary tract infections, anemia, heart failure, kidney failure, microbiology results, or surgeries on other parts of the body.

**Table 1:** Baseline characteristics of the hyperammonemia and non-hyperammonemia groups.

		**Hyperammonemia group *n* = 443**	**Non-hyperammonemia group *n* = 608**	***P***
**Baseline variables**				
Age, median [IQR]		61.8(50.0–72.2)	67.3(56.1–76.6)	<0.001
**Sex**, ***n*** **(%)**				
	Female	198(46.7)	227(37.3)	0.016
	Male	245(55.3)	381(62.7)	
**Vital signs, median [IQR]**				
	HR(bmp)	87.8(76.5–100.6)	86.8(77.5–98.0)	0.301
	SBP(mmHg)	116.8(106.2–130.5)	121.2(108.0–135.8)	0.011
	DBP(mmHg)	60.5(53.2–69.9)	60.0(54.0–67.6)	0.620
	MBP(mmHg)	77.6(70.0–87.0)	77.8(71.1–87.0)	0.468
	RR(bmp)	19.0(16.0–22.4)	18.8(16.1–21.6.0)	0.527
	T(°C)	36.9(36.4–37.4)	36.9(36.5–37.3)	0.918
**Laboratory parameters, median [IQR]**				
	ALT(IU/L)	25(14–49)	29(15–34.4)	0.439
	AST(IU/L)	29(17–50)	31.1(20.0–36.0)	0.551
	Cr(mg/dL)	1.2(0.8–2.2)	1.2(0.8–2.2)	0.632
	Glucose(mg/dL)	142(117–191)	149(117–186.8)	0.906
	Hemoglobin(g/dL)	10.0(8.8–11.7)	9.9(8.5–11.5)	0.159
	Platelet(X109/L)	185(121–259)	184.5(131.5–265.8)	0.254
	PTT	34.8(28.3–44.0)	33.5(27.7–44.0)	0.122
	INR	1.4(1.2–1.8)	1.3(1.2–1.8)	0.023
	PT	15.2(13.5–17.9)	14.8(13.3–17.9)	0.040
	BUN(mg/dL)	23(15–45)	25(16–43)	0.264
	WBC(X 109/L)	12.0(8.6–16.3)	11.5(8.3–15.8)	0.333
	Potassium_min(mmol/L)	3.8(3.5–4.2)	3.8(3.5–4.2)	0.705
	Potassium_max(mmol/L)	4.4(4.0–4.9)	4.4(4.0–4.9)	0.392
	Sodium_min(mmol/L)	137(134–140)	138(135–140)	0.210
	Sodium_max(mmol/L)	140(137–143)	140(138–143)	0.495
	Ammonia(μmol/L)	52(41–70)	23.5(18–29)	<0.001
**Disease type**, ***n*** **(%)**				
	Gastrointestinal bleeding	53(12.0)	69(11.3)	0.758
	Intestinal infection	45(10.2)	95(15.6)	0.010
	Urinary tract infection	120(27.1)	191(31.4)	0.129
	Sepsis	116(26.2)	160(26.3)	0.962
	Obesity	39(8.8)	29(4.8)	0.009
	Anemia	220(49.7)	381(62.7)	<0.001
	Heart failure	230(51.9)	405(66.7)	<0.001
	Kidney failure	275(62.1)	407(66.9)	0.103
	Disorders of urea cycle metabolism	4(0.9)	0(0)	<0.001
	Epilepsy	84(19.0)	66(10.9)	<0.001
**Microbiology type**, ***n*** **(%)**				
	Clostridium difficile	33(7.4)	54(8.9)	0.405
	Staph aureus	114(25.7)	200(32.9)	0.012
	Gram-negative bacteria	114(25.7)	162(26.6)	0.740
	Enterococcus	114(25.7)	178(29.3)	0.205
	Yeast	170(38.4)	221(36.3)	0.502
	Klebslella	62(14.0)	88(14.5)	0.827
	Acinetobacter baumannii	11(2.5)	16(2.6)	0.881
**Drug type**, ***n*** **(%)**				
	Corticosteroids	273(61.6)	270(44.4)	<0.001
	Carbamazepine	36(8.1)	14(3.1)	<0.001
	Valproic acid	43(9.7)	36(5.9)	0.022
**Surgery type**, ***n*** **(%)**				
	Bariatric surgery	1(0.2)	3(0.5)	0.486
	Gastric surgery	1(0.2)	0(0)	0.171
	Thoracic or abdominal surgery	11(2.5)	16(2.6)	0.881
	Cardiovascular surgery	32(7.2)	99(16.3)	<0.001
	Neuro surgery	53(12.0)	94(15.5)	0.107
	Orthopedic surgery	20(4.5)	15(2.5)	0.068
	Vascular surgery	19(4.3)	53(8.7)	0.005
**Score system, median [IQR]**				
	SAPSII	37(27–48)	37.5(29–48)	0.258
	qSOFA	2.0(1.0–2.0)	2.0(1.0–2.0)	0.717
	SOFA	5.0(3.0–7.0)	5.0(3.0–7.0)	0.204
	GCS	14(13–15)	14(11–15)	0.083

*ALT, alanine aminotransferase; AST, aspartate aminotransferase; Cr, creatinine; SpO2, pulse oximetry; SBP, systolic pressure; DBP, diastolic pressure; SAPSII, simplified acute physiology score; HR, heart rate; RR, respiratory rate; WBC, white blood cell count; PTT, partial thromboplastin time; PT, prothrombin time; INR, international normalized ratio; BUN, blood urea nitrogen; T, temperature; qSOFA, quick sequential organ failure assessment; ICU, intensive care unit; SOFA, sequential organ failure assessment; GCS, Glasgow coma scale; max, maximum; min, minimum; P < 0.05 means significant different*.

### Patient Outcomes

Table 2 shows the outcomes for the hyperammonemia and hyperammonemia groups. There were no significant differences in patients with delirium, encephalopathy, cerebral edema, coma, the scores of disease severity, length of hospital or ICU stay. Patients in the non-hyperammonemia group had better outcomes (30 day mortality, 18.1 vs. 23.0%; renal replacement therapy, 4.8 vs. 7.4%) than the hyperammonemia group, but, there were no significant differences in patient 90 day mortality or 1 year mortality (see Table 2, Supplementary Materials 2, 3 for more detail).

**Table 2:** Outcomes of the hyperammonemia and non-hyperammonemia groups.

**Outcome**	**Hyperammonemia group *n* = 443**	**Non-hyperammonemia group *n* = 608**	***p*-values**
Mechanical ventilation, *n* (%)	250(56.4)	326(53.6)	0.365
Renal replacement therapy, *n* (%)	33(7.4)	29(4.8)	0.055
**Vasopressors**, ***n*** **(%)**			
Norepinephrine	100(22.6)	122(20.1)	0.325
Dopamine	42(9.5)	50(8.2)	0.476
Epinephrine	13(3.0)	23(3.8)	0.455
Delirium, *n* (%)	60(13.5)	98(16.1)	0.249
Encephalopathy, *n* (%)	111(25.1)	154(25.3)	0.920
Cerebral edema, *n* (%)	10(2.3)	18(3.0)	0.485
Coma, *n* (%)	41(9.3)	64(10.5)	0.479
**Length of stay, median [IQR]**			
In ICU	3.9(1.8–10.8)	3.5(1.8–9.0)	0.328
In hospital	12(6–22)	12(6–23)	0.382
**Mortality**, ***n*** **(%)**			
30 day	102(23.0)	110(18.1)	0.049
90 day	141(31.8)	178(29.3)	0.643
1 year	190(42.9)	248(40.8)	0.495

*ICU, intensive care unit; p < 0.05 = statistically significant*.

### Ammonia Was an Independent Prognostic Predictor

**Figure 2:**
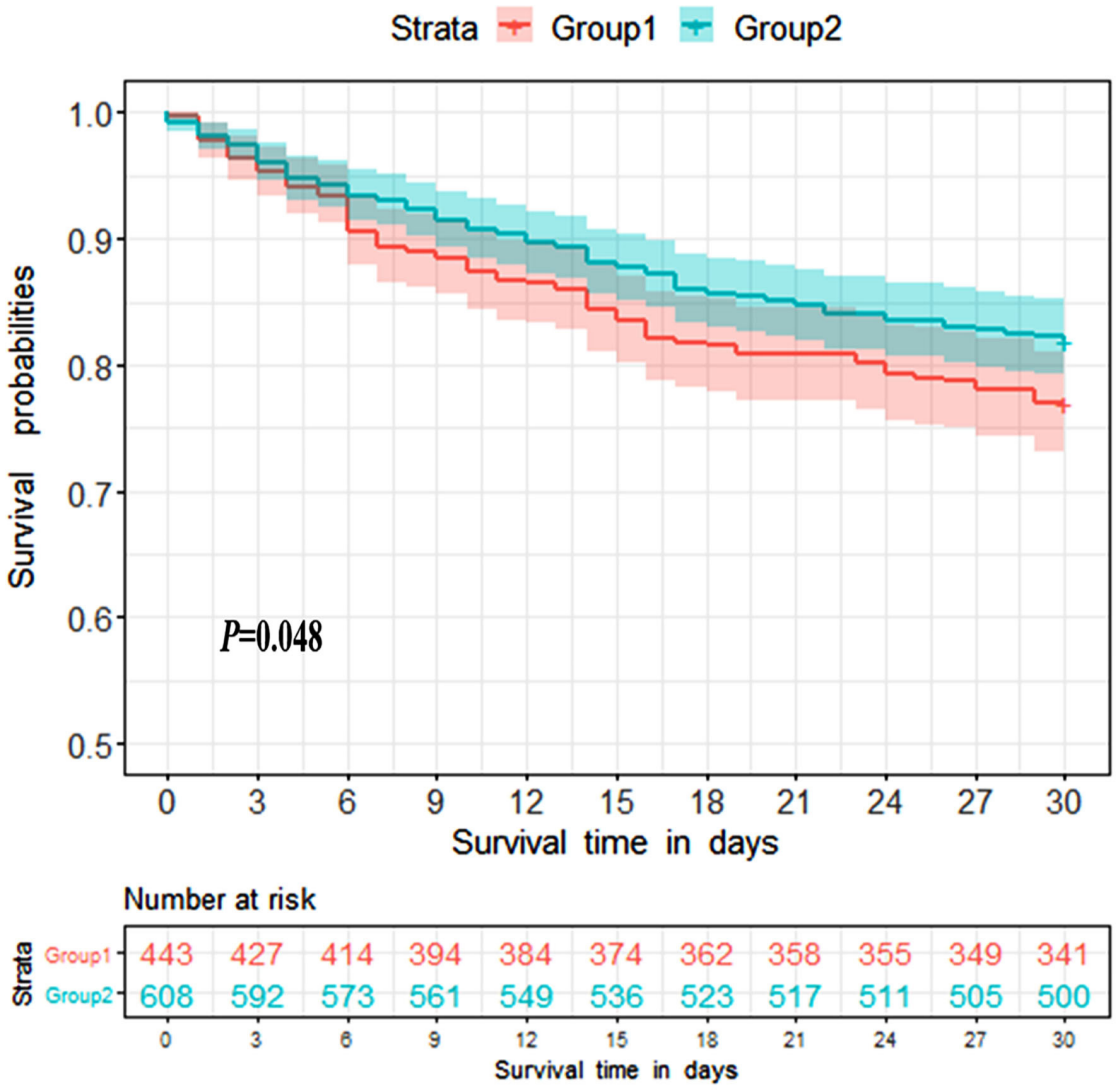
Kaplan-Meier 30 day survival curves for patients without liver disease. Group1 = Hyperammonemia group, Group2 = Non-hyperammonemia group.

Survival analysis was conducted to explore the impact of ammonia on prognosis. Patients in the non-hepatic hyperammonemia group had better short-term survival rates (30 day mortality) (Figure 2). Furthermore, we performed univariate analysis of the base-line variables (age, sex, first care unit) and laboratory tests. Age, sex, ALT, AST, Cr, BUN, glucose, hemoglobin, platelets, PTT, INR, PT, WBC, sodium, potassium, and ammonia were analyzed in the univariate analysis, and the factors significantly correlated with overall survival were adjusted for in the multivariate analysis. According to our results, ammonia, age, hemoglobin remained independent prognostic factors for NHH patients (*p* < 0.01 or *p* < 0.05) (see Table 3).

**Table 3:** Univariate and multivariate COX analysis of risk factors to 30 day mortality.

	**Univariate analysis**				**Multivariate analysis**			
	**RR**	**95.0% CI**		***p*-values**	**RR**	**95.0% CI**		***p*-values**
		**Lower**	**Upper**			**Lower**	**Upper**	
Age, median [IQR]	1.031	1.005	1.057	0.018	1.030	1.020	1.040	<0.001
Sex n (%)	1.251	0.799	1.959	0.328				
**Laboratory parameters, median [IQR]**								
ALT (IU/L)	1.007	1.002	1.013	0.008				
AST (IU/L)	1.008	1.004	1.014	0.005	1.008	1.001	1.014	0.018
Cr (mg/dL)	0.957	0.773	1.185	0.687				
BUN (mg/dL)	1.008	0.996	1.021	0.181				
Glucose (mg/dL)	1.003	0.998	1.008	0.316				
Hemoglobin (g/dL)	0.703	0.496	0.997	0.048	0.901	0.838	0.968	0.05
Platelet (X109/L)	1.000	0.997	1.003	0.867				
PTT	0.997	0.986	1.009	0.668				
INR	1.019	0.955	1.087	0.573				
PT	1.007	0.979	1.036	0.631				
WBC (X 109/L)	1.036	0.988	1.087	0.144				
Potassium_min (mmol/L)	2.379	1.169	4.841	0.017	1.120	0.898	1.396	0.315
Potassium_max (mmol/L)	1.266	0.906	1.769	0.166				
Sodium_min (mmol/L)	0.985	0.935	1.038	0.572				
Sodium_max (mmol/L)	1.006	0.940	1.075	0.871				
Ammonia (umol/L)	1.005	1.001	1.009	0.013	1.009	1.007	1.012	<0.001

*PT, prothrombin time; INR, international normalized ratio; BUN, blood urea nitrogen; min, minimum; max, maximum; RR, risk ratio; p < 0.05, statistically significant*.

## Discussion

In this study, we found the incidence of non-hepatic hyperammonemia is 42.2% in critical care patients. Patients with NHH had higher 30 day mortality than patients with non-hyperammonemia, and serum ammonia level was an independent predictor of mortality in patients without liver disease. Besides, we found obesity, orthopedic surgery, corticosteroids, carbamazepine, valproic acid, epilepsy, and disorders of urea cycle metabolism to be common risk factors for NHH.

In this study, we found the incidence of hyperammonemia in critical care patients without liver disease to be 42.2%. A recent prospective observational study with one hundred patients found, after excluding those patients with acute or chronic liver disease, hyperammonemia was observed in 40% of patients on their first day in the ICU and 60% by their third day in the ICU (5) A retrospective observational cohort study conducted on critically ill patients admitted to six ICUs found the lowest incidence of NHH in the literature at 8.6%, (4) while the highest recorded incidence was 73% (5) In previous studies, the largest sample size of non-hepatic hyperammonemia patients was still relatively small at 167 patients, (4) while the smallest sample size was 78 patients (18).

In our cohort, patients with hyperammonemia (23.0%) had a 30 day mortality significantly higher than patients without hyperammonemia (18.1%). Another study found patients with NHH had a hospital mortality rate of 64% and the other study in critically ill patients with NHH, ICU mortality was 50.6% and hospital mortality was 55.6% (6, 18). Based on previous studies and our own data, NHH not only has a high incidence but also a high mortality rate.

We found serum ammonia was an independent prognostic value for 30 day mortality in patients without a history of liver disease in both univariate and multivariate analyses. However, there are some small differences compared to two previous studies, (4, 19) both of these prior studies also showed NHH patients had a poor prognosis, a high in-hospital mortality rate, and a median serum ammonia level that was higher among non-survivors. Deaths were more likely in hyperammonemic patients who had greater illness severity, malignancy, solid organ transplantation, and cerebral edema, but, after adjustment, serum ammonia level was not associated with increased hospital or ICU mortality in these other studies. This may be due to the lack of post-discharge mortality statistics in these studies. Our cohort analyzed the prognosis of patients' 30 day, 90 day, and even 1 year after ICU stay mortality. Interestingly, ammonia level was an independent risk factor only for short-term prognosis in patients with critical illness in the ICU and not associated with longer-term mortality. This finding further supports the idea that NHH can lead to poor outcomes for patients, and should be approached with concern by clinicians.

We found seven risk factors for elevated blood ammonia in our study, which we have sorted into three categories: diseases, medications, and surgeries.

In terms of diseases, we found that obesity, disorders of urea cycle metabolism, and epilepsy were associated with NHH. The incidence of obesity in hyperammonemia patients was twice that of non-hyperammonemia patients. Obesity is a known risk factor for NHH, and seems to be related to a higher protein diet in obese patients, resulting in an increased production of urea and an elevated blood ammonia level (20). The incidence of disorders of urea cycle metabolism in patients with hyperammonemia was 0.9%.

The urea cycle is a metabolic pathway for the disposal of excess nitrogen, in which ammonia is the common product. Nitrogen is essential for growth and life, but excessive ammonia leads to several life-threatening conditions. Ammonia is a by-product of amino acid metabolism, and ornithine transcarbamylase (OTC) is not only a key enzyme of the urea cycle, but is also required for metabolism and excretion of ammonia. OTC catalyzes the mitochondrial reaction of ornithine to produce citrulline. Deficiency of OTC leads to the formation of excess carbamoyl phosphate; some of which is excreted as an orotic acid. When this pathway is overwhelmed, hyperammonemia results. Low arginine levels have been demonstrated to lead OTC deficiencies and decreased OTC activity. Arginine can also be hydrolyzed to release urea and regenerate ornithine. In the cytosol, arginine is catalyzed by arginase and hydrolyzed to urea and ornithine (21, 22). Therefore, a lack or reduced activity of OTC or low levels of arginine cause urea cycle disorders, and increased ammonia levels. In this study, we attempted to fix a limitation of a previous study, which did not compare patients with hyperammonemia unrelated to liver disease with patients with normal ammonia levels (4).

Finally, the incidence of epilepsy was significantly higher in the hyperammonemia group than in the non-hyperammonemia group. We speculate that this association may be due to not only the extensive muscle contractions of epilepsy, but is also related to the treatment of epilepsy. Skeletal muscle usually consumes ammonia, but with activity it becomes a producer of ammonia, causing clinically significant hyperammonemia (23). Intense muscle activity causes severe lactic acidosis, and there is evidence that a rise in serum lactate in strenuous exercise is associated with hyperammonemia as well (19).

In our cohort, we found several mediations were associated with increased ammonia production. Specifically, several drugs used to treat epilepsy such as valproic acid and carbamazepine. Valproic acid in the hyperammonemia group patients (9.7%) was significantly higher than in non-hyperammonemia patients (5.9%). Carbamazepine was used in 8.1% of the hyperammonemia group patients, however, it was used in 3.1% of the non-hyperammonemia group patients. Valproic acid causes dose-dependent increases in plasma ammonia levels without causing overt liver injury (24) by inhibiting the activity of carbamoyl phosphate synthetase (25). Valproic acid directly inhibits the mitochondrial urea cycle enzyme, carbamoyl phosphate synthetize I, which leads to urea cycle disorders, and increased ammonia production as described above (26). Carbamazepine was found to reduce glutamine synthetase enzyme activity, which is responsible for the conversion of glutamate to glutamine, aiding in the detoxification of ammonia (27).

Corticosteroids have been widely used as adjunct therapy for many diseases for many decades, but in our cohort, we found corticosteroids to be a risk factor for NHH. The intestine is an important place to produce ammonia, and a recent mouse study found that corticosteroids induced ammonia release from the intestine. There appears to be a close relationship between epithelial sodium uptake and ammonia release (28), since one of the adverse effects of corticosteroids is hypernatremia, this effect of high sodium levels in the intestine may be the cause of an increased blood ammonia level in patients taking corticosteroids.

Certain surgeries were associated with an increased ammonia level in our study. Specifically, patients with hyperammonemia (4.5%) had a higher rate of recent orthopedic surgery than non-hyperammonemia (2.5%). Under normal conditions, skeletal muscle consumes ammonia to produce glutamine under the action of glutamine synthetase (29). When skeletal muscles are damaged, such as during orthopedic surgeries, glutamine synthetase activity is broken, and its consumption of ammonia disappears, thus making skeletal muscle a net ammonia producer (23). When skeletal muscle generates adenosine triphosphate, it can also generate a molecule of adenosine monophosphate (30). Adenosine monophosphate is deaminated to inosine monophosphate to maintain equilibrium for the upstream dephosphorylation reactions with a concurrent production of ammonia (31). Of note, there was no association with bariatric surgery in our study, which is inconsistent with previous findings (32). This may be related to the small number of patients undergoing bariatric surgery reported in the database used in this study.

Urea produced by the liver is transported into the colon, and it is hydrolyzed by urease in the gut bacteria into ammonia (33). Urease can be produced by many different bacteria, and ureolytic activity is often observed in pathogenic bacteria (34). Previous research found *Klebisiella* species and *Pseudomonas aeruginosa* can cause an increase in ammonia production. It causes non-hepatic hyperammonemia encephalopathy (16, 35). They may be attributed to produce urease. Although our cohort found that the above bacteria are not risk factors for the occurrence of non-hepatic hyperammonemia, it is still worthy of our attention and further research.

## Limitations

A portion of the adult patients in the database were excluded due to a lack of vital signs or ICD 9 diagnostic codes, which may affect our conclusions. There were some patients missing certain values such as lactate, albumin, or bilirubin, which may be of interest to some readers. Our study is limited by its retrospective nature and the causal relationship between serum ammonia level and severity of illness cannot be determined. Further prospective follow-up study may help to address this shortcoming.

## Conclusion

NHH has a high incidence and mortality in critical-care patients, and blood ammonia level was an independent risk factor for 30 day mortality. Three types of risk factors associated with NHH were diseases (epilepsy, disorders of urea cycle metabolism, and obesity), drugs (valproic acid, carbamazepine, and corticosteroids) and orthopedic surgeries. Monitoring patients' serum ammonia levels is recommended not only for patients with liver disease but also those without liver problems, such as obesity and was diagnosed with disorders of urea cycle metabolism of patients, receiving orthopedic surgery, using corticosteroids, carbamazepine, valproic acid, epilepsy drugs.

## Data Availability Statement

All datasets generated for this study are included in the article/Supplementary Material.

## Ethics Statement

The studies involving human participants were reviewed and approved by the institutional review boards of the Massachusetts Institute of Technology and Beth Israel Deaconess Medical Center. Written informed consent for participation was not required for this study in accordance with the national legislation and the institutional requirements.

## Author Contributions

YL and LZ wrote the main manuscript text, included contributed to the conception, designed the work, and analyzed and interpreted data. JW, YG, XL, SY, HZ, and ZG collected the data regarding the paper. YL ensured that original data upon which the submission was based was preserved and retrievable for reanalysis approved data presentation as representative of the original data and foreseeing and minimized obstacles to the sharing of data described in the work. All authors read and approved the final manuscript.

## Conflict of Interest

The authors declare that the research was conducted in the absence of any commercial or financial relationships that could be construed as a potential conflict of interest.

## Abbreviations

HR: Heart rate (HR)
SBP: systolic blood pressure
DBP: diastolic blood pressure
RR: respiratory rate
T: temperature
ALT: alanine aminotransferase
AST: aspartate aminotransferase
PTT: partial thromboplastin time
INR: international normalized ratio
PT: prothrombin time
WBC: white blood cell count
BUN: blood urea nitrogen
Cr: creatinine
SAPSII: patients&#8217 simplified acute physiology score
qSOFA: quick sequential organ failure assessment
SOFA: sequential organ failure assessment
GCS: Glasgow coma scale
ICU: intensive care unit
NHH: non-hepatic hyperammonemia
MIMIC-III: Medical Information Mart for Intensive Care III
ICD-9: International Classification of Diseases, Ninth Revision
RR: risk ratio.
