# Supplementary material for: Prognostic Role of Ammonia in Critical Care Patients Without Known Hepatic Disease
Source: Front Med (Lausanne). 2020 Oct 22;7:589825. doi: 10.3389/fmed.2020.589825 (PMC7642587; doi:10.3389/fmed.2020.589825)
Supplement: Supplementary file 1 [file Table_1.DOCX]

| Supplementary materials 1 Exclude patients with acute and chronic liver disease from the MIMIC III database according to ICD9-codes | | | | | | | | | | | |
| --- | --- | --- | --- | --- | --- | --- | --- | --- | --- | --- | --- |
| ICD9-code | | Description |  |  |  |  |  |  |  |  |  |
| 700 |  | Hepatitis A with coma | |  |  |  |  |  |  |  |  |
| 0701 |  | Viral hepatitis A without mention of hepatic coma | | | | | | | |  |  |
| 07020 |  | Viral hepatitis B with hepatic coma, acute or unspecified, without mention of hepatitis delta | | | | | | | |  |  |
| 07021 |  | Viral hepatitis B with hepatic coma, acute or unspecified, with hepatitis delta | | | | | | |  |  |  |
| 07022 |  | Chronic viral hepatitis B with hepatic coma without hepatitis delta | | | | | |  |  |  |  |
| 07023 |  | Chronic viral hepatitis B with hepatic coma with hepatitis delta | | | | | | | | | |
| 07030 |  | Viral hepatitis B without mention of hepatic coma, acute or unspecified, without mention of hepatitis | | | | | | | | |  |
| 07031 |  | Viral hepatitis B without mention of hepatic coma, acute or unspecified, with hepatitis delta | | | | | | | |  |  |
| 07032 |  | Chronic viral hepatitis B without mention of hepatic coma without mention of hepatitis delta | | | | | | | |  |  |
| 07033 |  | Chronic viral hepatitis B without mention of hepatic coma with hepatitis delta | | | | | | |  |  |  |
| 07041 |  | Acute hepatitis C with hepatic coma | | | | | | | | | |
| 07042 |  | Hepatitis delta without mention of active hepatitis B disease with hepatic coma | | | | | | |  |  |  |
| 07043 |  | Hepatitis E with hepatic coma | | | | | | | | |  |
| 07044 |  | Chronic hepatitis C with hepatic coma | | | | | | | | |  |
| 07049 |  | Other specified viral hepatitis with hepatic coma | | | | | | | | |  |
| 07051 |  | Acute hepatitis C without mention of hepatic coma | | | | | | | | |  |
| 07052 |  | Hepatitis delta without mention of active hepatitis B disease or hepatic coma | | | | | | |  |  |  |
| 07053 |  | Hepatitis E without mention of hepatic coma | | | |  |  |  |  |  |  |
| 07054 |  | Chronic hepatitis C without mention of hepatic coma | | | | | | | |  |  |
| 07059 |  | Other specified viral hepatitis without mention of hepatic coma | | | | | | | |  |  |
| 0706 |  | Unspecified viral hepatitis with hepatic coma | | | |  |  |  |  |  |  |
| 07070 |  | Unspecified viral hepatitis C without hepatic coma | | | | | | | | |  |
| 07071 |  | Unspecified viral hepatitis C with hepatic coma | | | |  |  |  |  |  |  |
| 0709 |  | Unspecified viral hepatitis without mention of hepatic coma | | | | |  |  |  |  |  |
| 5712 |  | Alcoholic cirrhosis of liver | | | | | | |  |  |  |
| 5713 |  | Alcoholic liver damage, unspecified | | | | | | | |  |  |
| 57140 |  | Chronic hepatitis, unspecified | | | | | | | | | |
| 57141 |  | Chronic persistent hepatitis | | | | | | | |  |  |
| 57142 |  | Autoimmune hepatitis | |  |  |  |  |  |  |  |  |
| 57149 |  | Other chronic hepatitis | |  |  |  |  |  |  |  |  |
| 5715 |  | Cirrhosis of liver without mention of alcohol | | | |  |  |  |  |  |  |
| 5716 |  | Biliary cirrhosis | | | | | | |  |  |  |
| 5718 |  | Other chronic nonalcoholic liver disease | | | | | | | | | |
| 5719 |  | Unspecified chronic liver disease without mention of alcohol | | | | | | | |  |  |
| 5722 |  | Hepatic encephalopathy | | | | | | | | | |
| 5724 |  | Hepatorenal syndrome | |  |  |  |  |  |  |  |  |
| 5728 |  | Other sequelae of chronic liver disease | | | | | | |  |  |  |
| 5738 |  | Other specified disorders of liver | | |  |  |  |  |  |  |  |
| 5735 |  | Hepatopulmonary syndrome | | | | | | | | | |
| 5734 |  | Hepatic infarction | | | | | | | | | |
| 5733 |  | Hepatitis, unspecified | |  |  |  |  |  |  |  |  |
| 5732 |  | Hepatitis in other infectious diseases classified elsewhere | | | | |  |  |  |  |  |
| 5731 |  | Hepatitis in viral diseases classified elsewhere | | | |  |  |  |  |  |  |
| 5730 |  | Chronic passive congestion of liver | | |  |  |  |  |  |  |  |
| V0260 |  | Viral hepatitis carrier, unspecified | | |  |  |  |  |  |  |  |
| V0261 |  | Hepatitis B | carrier |  |  |  |  |  |  |  |  |
| V0262 |  | Hepatitis C | carrier |  |  |  |  |  |  |  |  |
| V0269 |  | Other viral hepatitis carrier | | | | |  |  |  |  |  |
| 86400 |  | Injury to liver without mention of open wound into cavity, unspecified injury | | | | | | |  |  |  |
| 86401 |  | Injury to liver without mention of open wound into cavity, hematoma and contusion | | | | | | |  |  |  |
| 86402 |  | Injury to liver without mention of open wound into cavity, laceration, minor | | | | | | |  |  |  |
| 86403 |  | Injury to liver without mention of open wound into cavity, laceration, moderate | | | | | | |  |  |  |
| 86404 |  | Injury to liver without mention of open wound into cavity, laceration, major | | | | | | |  |  |  |
| 86405 |  | Injury to liver without mention of open wound into cavity laceration, unspecified | | | | | | |  |  |  |
| 86409 |  | Other injury to liver without mention of open wound into cavity | | | | | | |  |  |  |
| 86410 |  | Injury to liver with open wound into cavity, unspecified injury | | | | | | | |  |  |
| 4560 |  | Esophageal varices with bleeding | | |  |  |  |  |  |  |  |
| 4561 |  | Esophageal varices without mention of bleeding | | | | | | | |  |  |
| 45620 |  | Esophageal varices in diseases classified elsewhere, with bleeding | | | | | |  |  |  |  |
| 45621 |  | Esophageal varices in diseases classified elsewhere, without mention of bleeding | | | | | | |  |  |  |

**^[[1]](#footnote-1)^ Prognostic role of ammonia in critical care patients without known hepatic disese: A cohort study from the MIMIC-III database**

Lina Zhao^1^, Joseph Harold Walline^2^, Yanxia Gao^3^, Xin Lu^1^, Shiyuan Yu^1^, Zengzheng Ge^1^, Huadong Zhu^1^, Yi Li^1*^

^1^ Emergency Department, Peking Union Medical College Hospital, Peking Union Medical College, Chinese Academy of Medical Sciences, Beijing, China, 100730

^2^ Accident and Emergency Medicine Academic Unit, Prince of Wales Hospital, The Chinese University of Hong Kong, Hong Kong, China, 999077

^3^ Emergency Department, The First Affiliated Hospital of Zhengzhou University, Zhengzhou, China, 450052

***Corresponding author:**

Yi Li.PhD: billliyi@126.com; (86)013693109826, ORCID: 0000-0002-7158-3624.

Emergency Department, Peking Union Medical College Hospital, Peking Union Medical College, Chinese Academy of Medical Sciences, Beijing, China, 100730

**Email address and telephone number:**

Lina Zhao. PhD: 15648833413@163.com; (86)015648833413

Joseph Harold Walline. PhD: jwallinemd@gmail.com；(86)013522414351

Yanxia Gao.MD: gaoyanxiazzu@163.com; (86)015136161660

Xin Lu.MD: luxin61@126.com; (86)018366116336

Shiyuan Yu.MD: 362384870@qq.com; (86)018904015983

Zengzheng Ge.MD：zengzhengge@126.com; (86)013192687634

Huadong Zhu.MD: zhuhuadong1970@126.com. (86)013910696435

1. [↑](#footnote-ref-1)
