# Supplementary material for: Prognostic Role of Ammonia in Critical Care Patients Without Known Hepatic Disease
Source: Front Med (Lausanne). 2020 Oct 22;7:589825. doi: 10.3389/fmed.2020.589825 (PMC7642587; doi:10.3389/fmed.2020.589825)
Supplement: Supplementary file 2 [file Table_2.DOCX]

| Additional file S2 Type of disease and ICD9-codes | | |  |  |  |  |  |  |  |  |  |
| --- | --- | --- | --- | --- | --- | --- | --- | --- | --- | --- | --- |
| Disease | ICD9-Code | Description |  |  |  |  |  |  |  |  |  |
| Gastrointestinal bleeding |  |  |  |  |  |  |  |  |  |  |  |
|  | 5789 | Hemorrhage of gastrointestinal tract, unspecified | | | |  |  |  |  |  |  |
|  | 5780 | Hematemesis |  |  |  |  |  |  |  |  |  |
|  | 5781 | Blood in stool |  |  |  |  |  |  |  |  |  |
|  | 5693 | Hemorrhage of rectum and anus | |  |  |  |  |  |  |  |  |
|  | 4560 | Esophageal varices with bleeding | | |  |  |  |  |  |  |  |
|  | 45620 | Esophageal varices in diseases classified elsewhere, with bleeding | | | | | |  |  |  |  |
|  | 53100 | Acute gastric ulcer with hemorrhage, without mention of obstruction | | | | | |  |  |  |  |
|  | 53101 | Acute gastric ulcer with hemorrhage, with obstruction | | | |  |  |  |  |  |  |
|  | 53120 | Acute gastric ulcer with hemorrhage and perforation, without mention of obstruction | | | | | | |  |  |  |
|  | 53121 | Acute gastric ulcer with hemorrhage and perforation, with obstruction | | | | | |  |  |  |  |
|  | 53300 | Acute peptic ulcer of unspecified site with hemorrhage, without mention of obstruction | | | | | | |  |  |  |
|  | 53320 | Acute peptic ulcer of unspecified site with hemorrhage and perforation, without mention of obstruction | | | | | | | |  |  |
|  | 53321 | Acute peptic ulcer of unspecified site with hemorrhage and perforation, with obstruction | | | | | | | |  |  |
|  | 53200 | Acute duodenal ulcer with hemorrhage, without mention of obstruction | | | | | |  |  |  |  |
|  | 53201 | Acute duodenal ulcer with hemorrhage, with obstruction | | | | |  |  |  |  |  |
|  | 53220 | Acute duodenal ulcer with hemorrhage and perforation, without mention of obstruction | | | | | | | |  |  |
|  | 53221 | Acute duodenal ulcer with hemorrhage and perforation, with obstruction | | | | | |  |  |  |  |
|  | 53400 | Acute gastrojejunal ulcer with hemorrhage, without mention of obstruction | | | | | |  |  |  |  |
|  | 53401 | Acute gastrojejunal ulcer, with hemorrhage, with obstruction | | | | |  |  |  |  |  |
|  | 53420 | Acute gastrojejunal ulcer with hemorrhage and perforation, without mention of obstruction | | | | | | | |  |  |
|  | 53421 | Acute gastrojejunal ulcer with hemorrhage and perforation, with obstruction | | | | | |  |  |  |  |
|  | 53501 | Acute gastritis, with hemorrhage | |  |  |  |  |  |  |  |  |
| Intestinal infection |  |  |  |  |  |  |  |  |  |  |  |
|  | 845 | Intestinal infection due to Clostridium difficile | | | |  |  |  |  |  |  |
|  | 847 | Intestinal infection due to other gram-negative bacteria | | | | |  |  |  |  |  |
|  | 88 | Intestinal infection due to other organism, not elsewhere classified | | | | | |  |  |  |  |
|  | 90 | Infectious colitis, enteritis, and gastroenteritis | | | |  |  |  |  |  |  |
|  | 93 | Diarrhea of presumed infectious origin | | |  |  |  |  |  |  |  |
|  | 56081 | Intestinal or peritoneal adhesions with obstruction (postoperative) (postinfection) | | | | | | |  |  |  |
|  | 56982 | Ulceration of intestine | |  |  |  |  |  |  |  |  |
|  | 56983 | Perforation of intestine | |  |  |  |  |  |  |  |  |
| Urinary tract infection |  |  |  |  |  |  |  |  |  |  |  |
|  | 5990 | Urinary tract infection | |  |  |  |  |  |  |  |  |
| Sepsis |  |  |  |  |  |  |  |  |  |  |  |
|  | 99591 | sepsis |  |  |  |  |  |  |  |  |  |
|  | 99592 | Severe sepsis |  |  |  |  |  |  |  |  |  |
|  | 78552 | Septic shock |  |  |  |  |  |  |  |  |  |
| Obesity |  |  |  |  |  |  |  |  |  |  |  |
|  | 27800 | Obesity, unspecified |  |  |  |  |  |  |  |  |  |
|  | 27801 | Morbid obesity |  |  |  |  |  |  |  |  |  |
|  | 27802 | Overweight |  |  |  |  |  |  |  |  |  |
|  | 27803 | Obesity hypoventilation syndrome | | |  |  |  |  |  |  |  |
| Anemia |  |  |  |  |  |  |  |  |  |  |  |
|  | 2800 | Iron deficiency anemia secondary to blood loss (chronic) | | | | |  |  |  |  |  |
|  | 2808 | Other specified iron deficiency anemias | | |  |  |  |  |  |  |  |
|  | 2809 | Iron deficiency anemia, unspecified | | |  |  |  |  |  |  |  |
|  | 2810 | Pernicious anemia |  |  |  |  |  |  |  |  |  |
|  | 2811 | Other vitamin B12 deficiency anemia | | |  |  |  |  |  |  |  |
|  | 2812 | Folate-deficiency anemia | |  |  |  |  |  |  |  |  |
|  | 2818 | Anemia associated with other specified nutritional deficiency | | | | |  |  |  |  |  |
|  | 2819 | Unspecified deficiency anemia | |  |  |  |  |  |  |  |  |
|  | 2822 | Anemias due to disorders of glutathione metabolism | | | |  |  |  |  |  |  |
|  | 2829 | Hereditary hemolytic anemia, unspecified | | |  |  |  |  |  |  |  |
|  | 2830 | Autoimmune hemolytic anemias | |  |  |  |  |  |  |  |  |
|  | 2839 | Acquired hemolytic anemia, unspecified | | |  |  |  |  |  |  |  |
|  | 2849 | Aplastic anemia, unspecified | |  |  |  |  |  |  |  |  |
|  | 2851 | Acute posthemorrhagic anemia | |  |  |  |  |  |  |  |  |
|  | 2858 | Other specified anemias | |  |  |  |  |  |  |  |  |
|  | 2859 | Anemia, unspecified |  |  |  |  |  |  |  |  |  |
|  | 7765 | Congenital anemia |  |  |  |  |  |  |  |  |  |
|  | 7766 | Anemia of prematurity | |  |  |  |  |  |  |  |  |
|  | 28489 | Other specified aplastic anemias | |  |  |  |  |  |  |  |  |
|  | 28521 | Anemia in chronic kidney disease | | |  |  |  |  |  |  |  |
|  | 28522 | Anemia in neoplastic disease | |  |  |  |  |  |  |  |  |
|  | 28529 | Anemia of other chronic disease | |  |  |  |  |  |  |  |  |
| Heart failure |  |  |  |  |  |  |  |  |  |  |  |
|  | 4280 | Congestive heart failure, unspecified | | |  |  |  |  |  |  |  |
|  | 4281 | Left heart failure |  |  |  |  |  |  |  |  |  |
|  | 42830 | Diastolic heart failure, unspecified | | |  |  |  |  |  |  |  |
|  | 42831 | Acute diastolic heart failure | |  |  |  |  |  |  |  |  |
|  | 42832 | Chronic diastolic heart failure | |  |  |  |  |  |  |  |  |
|  | 42833 | Acute on chronic diastolic heart failure | | |  |  |  |  |  |  |  |
|  | 42840 | Combined systolic and diastolic heart failure, unspecified | | | | |  |  |  |  |  |
|  | 42841 | Acute combined systolic and diastolic heart failure | | | |  |  |  |  |  |  |
|  | 42842 | Chronic combined systolic and diastolic heart failure | | | |  |  |  |  |  |  |
|  | 42843 | Acute on chronic combined systolic and diastolic heart failure | | | | |  |  |  |  |  |
|  | 39891 | Rheumatic heart failure (congestive) | | |  |  |  |  |  |  |  |
| Kidney failure |  |  |  |  |  |  |  |  |  |  |  |
|  | 5845 | Acute kidney failure with lesion of tubular necrosis | | | |  |  |  |  |  |  |
|  | 5846 | Acute kidney failure with lesion of renal cortical necrosis | | | | |  |  |  |  |  |
|  | 5848 | Acute kidney failure with other specified pathological lesion in kidney | | | | | |  |  |  |  |
|  | 5849 | Acute kidney failure, unspecified | | |  |  |  |  |  |  |  |
|  | 5852 | Chronic kidney disease, Stage II (mild) | | |  |  |  |  |  |  |  |
|  | 5853 | Chronic kidney disease, Stage III (moderate) | | | |  |  |  |  |  |  |
|  | 5854 | Chronic kidney disease, Stage IV (severe) | | |  |  |  |  |  |  |  |
|  | 5855 | Chronic kidney disease, Stage V | |  |  |  |  |  |  |  |  |
|  | 5856 | End stage renal disease | |  |  |  |  |  |  |  |  |
| Disorders of urea cycle metabolism | |  |  |  |  |  |  |  |  |  |  |
|  | 2706 | Disorders of urea cycle metabolism | | |  |  |  |  |  |  |  |
| Epilepsy |  |  |  |  |  |  |  |  |  |  |  |
|  | 34500 | Generalized nonconvulsive epilepsy, without mention of intractable epilepsy | | | | | | |  |  |  |
|  | 34501 | Generalized nonconvulsive epilepsy, with intractable epilepsy | | | | |  |  |  |  |  |
|  | 34510 | Generalized convulsive epilepsy, without mention of intractable epilepsy | | | | | |  |  |  |  |
|  | 34511 | Generalized convulsive epilepsy, with intractable epilepsy | | | | |  |  |  |  |  |
|  | 34540 | Localization-related epilepsy and epileptic syndromes with complex partial seizures, without mention of intractable epilepsy | | | | | | | |  |  |
|  | 34541 | Localization-related epilepsy and epileptic syndromes with complex partial seizures, with intractable epilepsy | | | | | | | |  |  |
|  | 34550 | Localization-related epilepsy and epileptic syndromes with simple partial seizures, without mention of intractable epilepsy | | | | | | | |  |  |
|  | 34551 | Localization-related epilepsy and epileptic syndromes with simple partial seizures, with intractable epilepsy | | | | | | | |  |  |
|  | 34570 | Epilepsia partialis continua, without mention of intractable epilepsy | | | | | |  |  |  |  |
|  | 34571 | Epilepsia partialis continua, with intractable epilepsy | | | |  |  |  |  |  |  |
|  | 34580 | Other forms of epilepsy and recurrent seizures, without mention of intractable epilepsy | | | | | | |  |  |  |
|  | 34581 | Other forms of epilepsy and recurrent seizures, with intractable epilepsy | | | | | |  |  |  |  |
|  | 34590 | Epilepsy, unspecified, without mention of intractable epilepsy | | | | |  |  |  |  |  |
|  | 34591 | Epilepsy, unspecified, with intractable epilepsy | | | |  |  |  |  |  |  |
|  | 3453 | Grand mal status |  |  |  |  |  |  |  |  |  |
