# Supplementary material for: Prognostic Role of Ammonia in Critical Care Patients Without Known Hepatic Disease
Source: Front Med (Lausanne). 2020 Oct 22;7:589825. doi: 10.3389/fmed.2020.589825 (PMC7642587; doi:10.3389/fmed.2020.589825)
Supplement: Supplementary file 3 [file Table_3.DOCX]

| Supplementary materials 3Outcome of patients in the hyperammonemia group and non-hyperammonemia group and ICD9-codes | | | | | | | | | | | | | | | | | | | | | | |  |  |  |  |  |  |  |  |  |
| --- | --- | --- | --- | --- | --- | --- | --- | --- | --- | --- | --- | --- | --- | --- | --- | --- | --- | --- | --- | --- | --- | --- | --- | --- | --- | --- | --- | --- | --- | --- | --- |
|  |  | ICD9-code | | | Description | | |  | |  | |  | |  | |  | |  |  |  |  |  |  |  |  |  |  |  |  |  |  |
| Delirium |  |  |  |  | |  | |  | |  | |  | |  | |  | |  |  |  |  |  |  |  |  |  |  |  |  |  |  |
|  |  | 29041 |  | Vascular dementia, with delirium | | | | | | | | |  | |  | |  | |  |  |  |  |  |  |  |  |  |  |  |  |  |
|  |  | 29043 |  | Vascular dementia, with depressed mood | | | | | | | | |  | |  | |  | |  |  |  |  |  |  |  |  |  |  |  |  |  |
|  |  | 29281 |  | Drug-induced delirium | | | | | | |  | |  | |  | |  | |  |  |  |  |  |  |  |  |  |  |  |  |  |
|  |  | 2910 |  | Alcohol withdrawal delirium | | | | | | |  | |  | |  | |  | |  |  |  |  |  |  |  |  |  |  |  |  |  |
|  |  | 2930 |  | Delirium due to conditions classified elsewhere | | | | | | | | | | |  | |  | |  |  |  |  |  |  |  |  |  |  |  |  |  |
|  |  |  |  |  | | |  | |  | |  | |  | |  | |  | |  |  |  |  |  |  |  |  |  |  |  |  |  |
| Encephalopathy |  | 4372 |  | Hypertensive encephalopathy | | | | | | |  | |  | |  | |  | |  |  |  |  |  |  |  |  |  |  |  |  |  |
|  |  | 34982 |  | Toxic encephalopathy | | | | | | |  | |  | |  | |  | |  |  |  |  |  |  |  |  |  |  |  |  |  |
|  |  | 34831 |  | Metabolic encephalopathy | | | | | | |  | |  | |  | |  | |  |  |  |  |  |  |  |  |  |  |  |  |  |
|  |  | 34830 |  | Encephalopathy, unspecified | | | | | | |  | |  | |  | |  | |  |  |  |  |  |  |  |  |  |  |  |  |  |
|  |  | 34839 |  | Other encephalopathy | | | | | | |  | |  | |  | |  | |  |  |  |  |  |  |  |  |  |  |  |  |  |
| Coma |  |  |  |  | | |  | |  | |  | |  | |  | |  | |  |  |  |  |  |  |  |  |  |  |  |  |  |
|  |  | 8500 |  | Concussion with no loss of consciousness | | | | | | | | |  | |  | |  | |  |  |  |  |  |  |  |  |  |  |  |  |  |
|  |  | 8505 |  | Concussion with loss of consciousness of unspecified duration | | | | | | | | | | | | |  | |  |  |  |  |  |  |  |  |  |  |  |  |  |
|  |  | 25031 |  | Diabetes with other coma, type I [juvenile type], not stated as uncontrolled | | | | | | | | | | | | | | |  |  |  |  |  |  |  |  |  |  |  |  |  |
|  |  | 25032 |  | Diabetes with other coma, type II or unspecified type, uncontrolled | | | | | | | | | | | | | | |  |  |  |  |  |  |  |  |  |  |  |  |  |
|  |  | 25033 |  | Diabetes with other coma, type I [juvenile type], uncontrolled | | | | | | | | | | | | |  | |  |  |  |  |  |  |  |  |  |  |  |  |  |
|  |  | 80021 |  | Closed fracture of vault of skull with subarachnoid, subdural, and extradural hemorrhage, with no loss of conscious | | | | | | | | | | | | | | | | | | |  |  |  |  |  |  |  |  |  |
|  |  | 80022 |  | Closed fracture of vault of skull with subarachnoid, subdural, and extradural hemorrhage, with brief [less than one hour] loss of consciousness | | | | | | | | | | | | | | | | | | | | | |  |  |  |  |  |  |
|  |  | 80026 |  | Closed fracture of vault of skull with subarachnoid, subdural, and extradural hemorrhage, with loss of consciousness of unspecified duration | | | | | | | | | | | | | | | | | | | | |  |  |  |  |  |  |  |
|  |  | 80071 |  | Open fracture of vault of skull with subarachnoid, subdural, and extradural hemorrhage, with no loss of consciousness | | | | | | | | | | | | | | | | | | |  |  |  |  |  |  |  |  |  |
|  |  | 80101 |  | Closed fracture of base of skull without mention of intra cranial injury, with no loss of consciousness | | | | | | | | | | | | | | | | | |  |  |  |  |  |  |  |  |  |  |
|  |  | 80106 |  | Closed fracture of base of skull without mention of intra cranial injury, with loss of consciousness of unspecified duration | | | | | | | | | | | | | | | | | | | |  |  |  |  |  |  |  |  |
|  |  | 80113 |  | Closed fracture of base of skull with cerebral laceration and contusion, with moderate [1-24 hours] loss of consciousness | | | | | | | | | | | | | | | | | | | |  |  |  |  |  |  |  |  |
|  |  | 80116 |  | Closed fracture of base of skull with cerebral laceration and contusion, with loss of consciousness of unspecified duration | | | | | | | | | | | | | | | | | | | |  |  |  |  |  |  |  |  |
|  |  | 80121 |  | Closed fracture of base of skull with subarachnoid, subdural, and extradural hemorrhage, with no loss of consciousness | | | | | | | | | | | | | | | | | | |  |  |  |  |  |  |  |  |  |
|  |  | 80122 |  | Closed fracture of base of skull with subarachnoid, subdural, and extradural hemorrhage, with brief [less than one hour] loss of consciousness | | | | | | | | | | | | | | | | | | | | | |  |  |  |  |  |  |
|  |  | 80126 |  | Closed fracture of base of skull with subarachnoid, subdural, and extradural hemorrhage, with loss of consciousness of unspecified duration | | | | | | | | | | | | | | | | | | | | |  |  |  |  |  |  |  |
|  |  | 80136 |  | Closed fracture of base of skull with other and unspecified intracranial hemorrhage, with loss of consciousness of unspecified | | | | | | | | | | | | | | | | | | | |  |  |  |  |  |  |  |  |
|  |  | 80312 |  | Other closed skull fracture with cerebral laceration and contusion, with brief [less than one hour] loss of consciousness | | | | | | | | | | | | | | | | | | |  |  |  |  |  |  |  |  |  |
|  |  | 80321 |  | Other closed skull fracture with subarachnoid, subdural, and extradural hemorrhage, with no loss of consciousness | | | | | | | | | | | | | | | | | | |  |  |  |  |  |  |  |  |  |
|  |  | 80325 |  | Other closed skull fracture with subarachnoid, subdural, and extradural hemorrhage, with prolonged [more than 24 hours] loss of consciousness, without return to pre-existing conscious level | | | | | | | | | | | | | | | | | | | | | | | | | |  |  |
|  |  | 80422 |  | Closed fractures involving skull or face with other bones with subarachnoid, subdural, and extradural hemorrhage, with brief [less than one hour] loss of consciousness | | | | | | | | | | | | | | | | | | | | | | | |  |  |  |  |
|  |  | 85011 |  | Concussion, with loss of consciousness of 30 minutes or less | | | | | | | | | | | | |  | |  |  |  |  |  |  |  |  |  |  |  |  |  |
|  |  | 85105 |  | Cortex (cerebral) contusion without mention of open intracranial wound, with prolonged [more than 24 hours] loss of consciousness without return to pre-existing conscious level | | | | | | | | | | | | | | | | | | | | | | | | |  |  |  |
|  |  | 85109 |  | Cortex (cerebral) contusion without mention of open intracranial wound, with prolonged [more than 24 hours] loss of consciousness without return to pre-existing conscious level | | | | | | | | | | | | | | | | | | | | | | | | |  |  |  |
|  |  | 85141 |  | Cerebellar or brain stem contusion without mention of open intracranial wound, with no loss of consciousness | | | | | | | | | | | | | | | | | | |  |  |  |  |  |  |  |  |  |
|  |  | 85145 |  | Cerebellar or brain stem contusion without mention of open intracranial wound, with prolonged [more than 24 hours] loss of consciousness without return to pre-existing conscious level | | | | | | | | | | | | | | | | | | | | | | | | | |  |  |
|  |  | 85146 |  | Cerebellar or brain stem contusion without mention of open intracranial wound, with loss of consciousness of unspecified duration | | | | | | | | | | | | | | | | | | | | |  |  |  |  |  |  |  |
|  |  | 85181 |  | Other and unspecified cerebral laceration and contusion, without mention of open intracranial wound, with no loss of consciousness | | | | | | | | | | | | | | | | | | | | |  |  |  |  |  |  |  |
|  |  | 85182 |  | Other and unspecified cerebral laceration and contusion, without mention of open intracranial wound, with brief [less than one hour] loss of consciousness | | | | | | | | | | | | | | | | | | | | | | |  |  |  |  |  |
|  |  | 85185 |  | Other and unspecified cerebral laceration and contusion, without mention of open intracranial wound, with prolonged [more than 24 hours] loss of consciousness without return to pre-existing conscious level | | | | | | | | | | | | | | | | | | | | | | | | | | | |
|  |  | 85186 |  | Other and unspecified cerebral laceration and contusion, without mention of open intracranial wound, with loss of consciousness of unspecified duration | | | | | | | | | | | | | | | | | | | | | | |  |  |  |  |  |
|  |  | 85201 |  | Subarachnoid hemorrhage following injury without mention of open intracranial wound, with no loss of consciousness | | | | | | | | | | | | | | | | | | |  |  |  |  |  |  |  |  |  |
|  |  | 85202 |  | Subarachnoid hemorrhage following injury without mention of open intracranial wound, with brief [less than one hour] loss of consciousness | | | | | | | | | | | | | | | | | | | | | |  |  |  |  |  |  |
|  |  | 85205 |  | Subarachnoid hemorrhage following injury without mention of open intracranial wound, with prolonged [more than 24 hours] loss of consciousness without return to pre-existing conscious level | | | | | | | | | | | | | | | | | | | | | | | | | |  |  |
|  |  | 85206 |  | Subarachnoid hemorrhage following injury without mention of open intracranial wound, with loss of consciousness of unspecified duration | | | | | | | | | | | | | | | | | | | | |  |  |  |  |  |  |  |
|  |  | 85216 |  | Subarachnoid hemorrhage following injury with open intracranial wound, with loss of consciousness of unspecified duration | | | | | | | | | | | | | | | | | | | |  |  |  |  |  |  |  |  |
|  |  | 85221 |  | Subdural hemorrhage following injury without mention of open intracranial wound, with no loss of consciousness | | | | | | | | | | | | | | | | | | |  |  |  |  |  |  |  |  |  |
|  |  | 85222 |  | Subdural hemorrhage following injury without mention of open intracranial wound, with brief [less than one hour] loss of consciousness | | | | | | | | | | | | | | | | | | | | |  |  |  |  |  |  |  |
|  |  | 85225 |  | Subdural hemorrhage following injury without mention of open intracranial wound, with prolonged [more than 24 hours] loss of consciousness without return to pre-existing conscious level | | | | | | | | | | | | | | | | | | | | | | | | | |  |  |
|  |  | 85226 |  | Subdural hemorrhage following injury without mention of open intracranial wound, with loss of consciousness of unspecified duration | | | | | | | | | | | | | | | | | | | | |  |  |  |  |  |  |  |
|  |  | 85241 |  | Extradural hemorrhage following injury without mention of open intracranial wound, with no loss of consciousness | | | | | | | | | | | | | | | | | | |  |  |  |  |  |  |  |  |  |
|  |  | 85302 |  | Other and unspecified intracranial hemorrhage following injury without mention of open intracranial wound, with brief [less than one hour] loss of consciousness | | | | | | | | | | | | | | | | | | | | | | |  |  |  |  |  |
|  |  | 85301 |  | Other and unspecified intracranial hemorrhage following injury without mention of open intracranial wound, with no loss of consciousness | | | | | | | | | | | | | | | | | | | | |  |  |  |  |  |  |  |
|  |  | 85305 |  | Other and unspecified intracranial hemorrhage following injury without mention of open intracranial wound, with prolonged [more than 24 hours] loss of consciousness without return to pre-existing conscious level | | | | | | | | | | | | | | | | | | | | | | | | | | | |
|  |  | 85306 |  | Other and unspecified intracranial hemorrhage following injury without mention of open intracranial wound, with loss of consciousness | | | | | | | | | | | | | | | | | | | | |  |  |  |  |  |  |  |
|  |  | 85402 |  | Intracranial injury of other and unspecified nature without mention of open intracranial wound, with brief [less than one hour] loss of consciousness | | | | | | | | | | | | | | | | | | | | | |  |  |  |  |  |  |
|  |  | 85406 |  | Intracranial injury of open intracranial wound, with loss of consciousness of unspecified duration | | | | | | | | | | | | | | | | |  |  |  |  |  |  |  |  |  |  |  |
|  |  | 78001 |  | Coma | | |  | |  | |  | |  | |  | |  | |  |  |  |  |  |  |  |  |  |  |  |  |  |
|  |  | 78003 |  | Persistent vegetative state | | | | | | |  | |  | |  | |  | |  |  |  |  |  |  |  |  |  |  |  |  |  |

**Prognostic role of ammonia in critical care patients without known hepatic disese: A cohort study from the MIMIC-III database**

Lina Zhao^1^, Joseph Harold Walline^2^, Yanxia Gao^3^, Xin Lu^1^, Shiyuan Yu^1^, Zengzheng Ge^1^, Huadong Zhu^1^, Yi Li^1*^

^1^ Emergency Department, Peking Union Medical College Hospital, Peking Union Medical College, Chinese Academy of Medical Sciences, Beijing, China, 100730

^2^ Accident and Emergency Medicine Academic Unit, Prince of Wales Hospital, The Chinese University of Hong Kong, Hong Kong, China, 999077

^3^ Emergency Department, The First Affiliated Hospital of Zhengzhou University, Zhengzhou, China, 450052

***Corresponding author:**

Yi Li.PhD: billliyi@126.com; (86)013693109826, ORCID: 0000-0002-7158-3624.

Emergency Department, Peking Union Medical College Hospital, Peking Union Medical College, Chinese Academy of Medical Sciences, Beijing, China, 100730

**Email address and telephone number:**

Lina Zhao. PhD: 15648833413@163.com; (86)015648833413

Joseph Harold Walline. PhD: jwallinemd@gmail.com；(86)013522414351

Yanxia Gao.MD: gaoyanxiazzu@163.com; (86)015136161660

Xin Lu.MD: luxin61@126.com; (86)018366116336

Shiyuan Yu.MD: 362384870@qq.com; (86)018904015983

Zengzheng Ge.MD：zengzhengge@126.com; (86)013192687634

Huadong Zhu.MD: zhuhuadong1970@126.com. (86)013910696435
