# Supplementary material for: Prognostic Role of Ammonia in Critical Care Patients Without Known Hepatic Disease
Source: Front Med (Lausanne). 2020 Oct 22;7:589825. doi: 10.3389/fmed.2020.589825 (PMC7642587; doi:10.3389/fmed.2020.589825)

是否将当前网页翻译成中文 网页翻译 中英对照 关闭

Data Profiling Report

- Basic Statistics
  - Raw Counts
  - Percentages
- Data Structure
- Missing Data Profile
- Univariate Distribution
  - Histogram
  - Bar Chart (by frequency)
  - QQ Plot
- Correlation Analysis
- Principal Component Analysis

Basic Statistics

Raw Counts

Name	Value
Rows	1,051
Columns	28
Discrete columns	1
Continuous columns	27
All missing columns	0
Missing observations	702
Complete Rows	807
Total observations	29,428
Memory allocation	187.3 Kb

Percentages

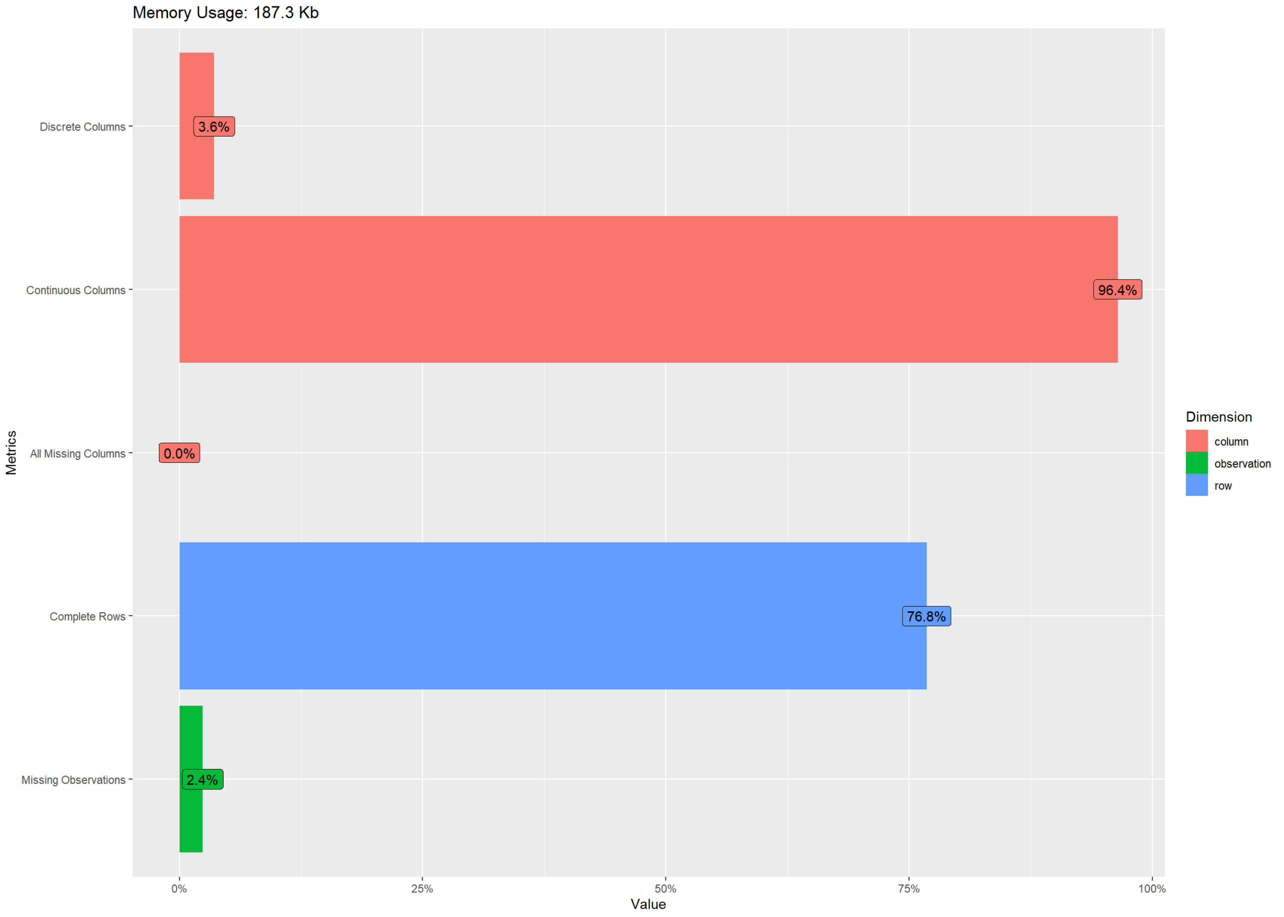

Data Structure

是否将当前网页翻译成中文 网页翻译 中英对照 关闭

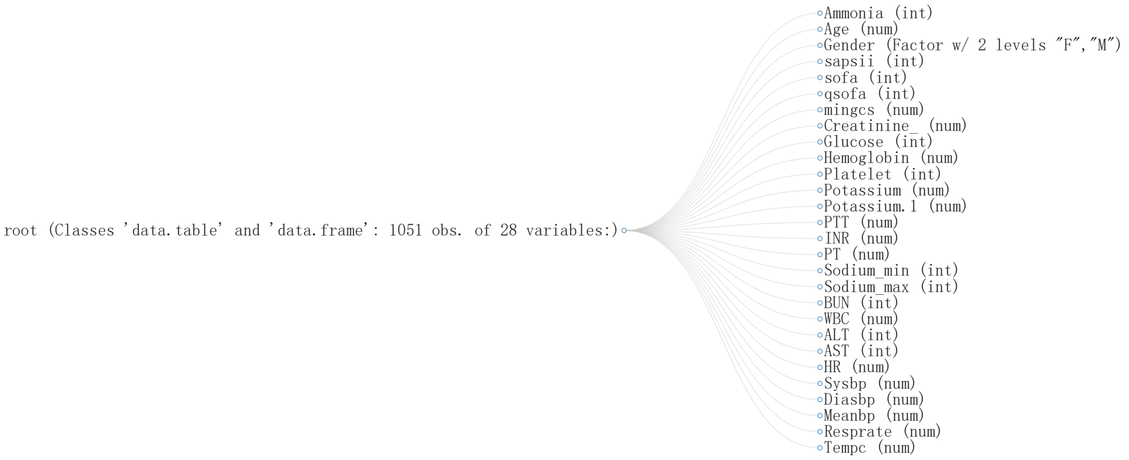

Missing Data Profile

是否将当前网页翻译成中文 网页翻译 中英对照 关闭

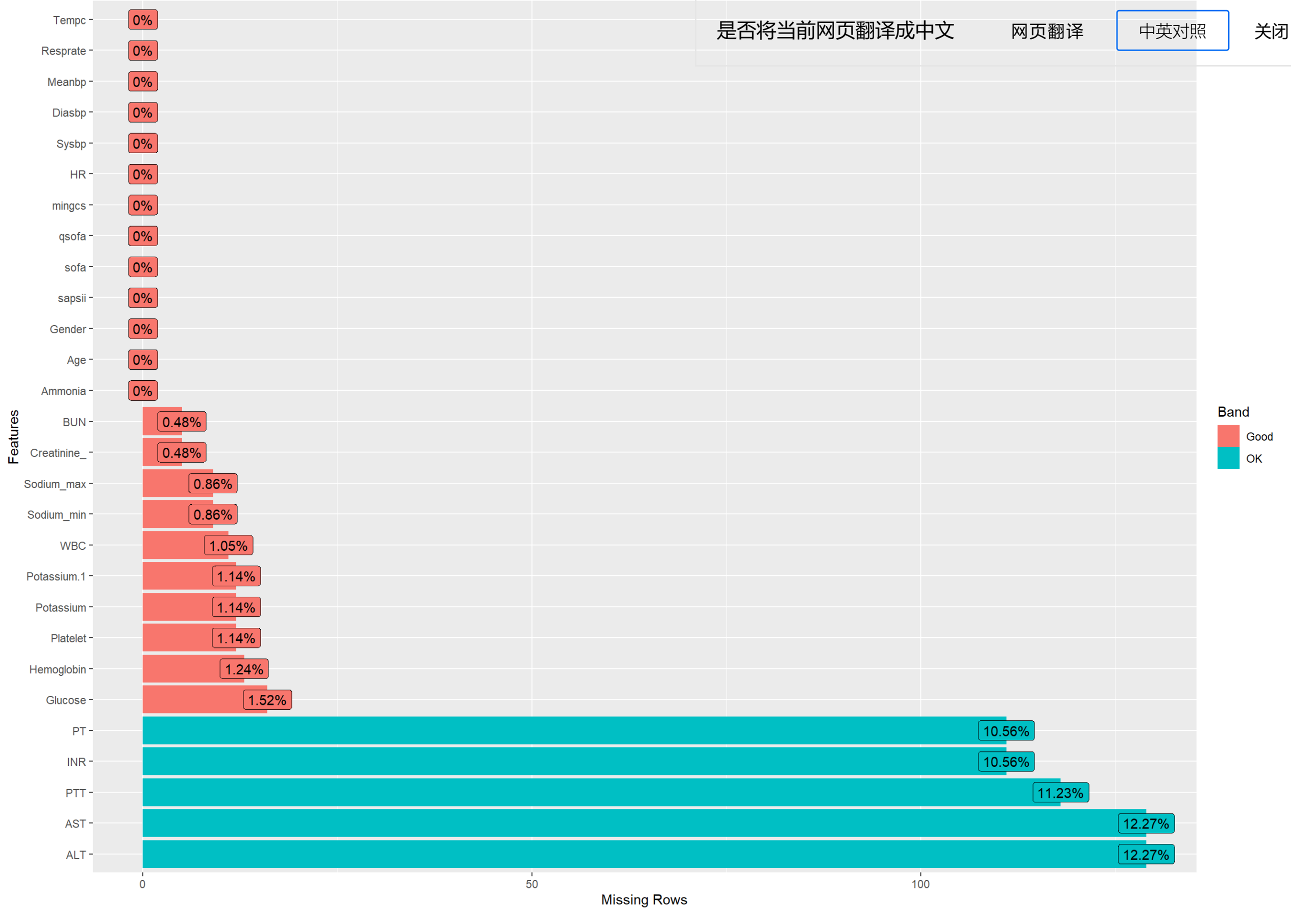

Univariate Distribution

Histogram

是否将当前网页翻译成中文 网页翻译 中英对照 关闭

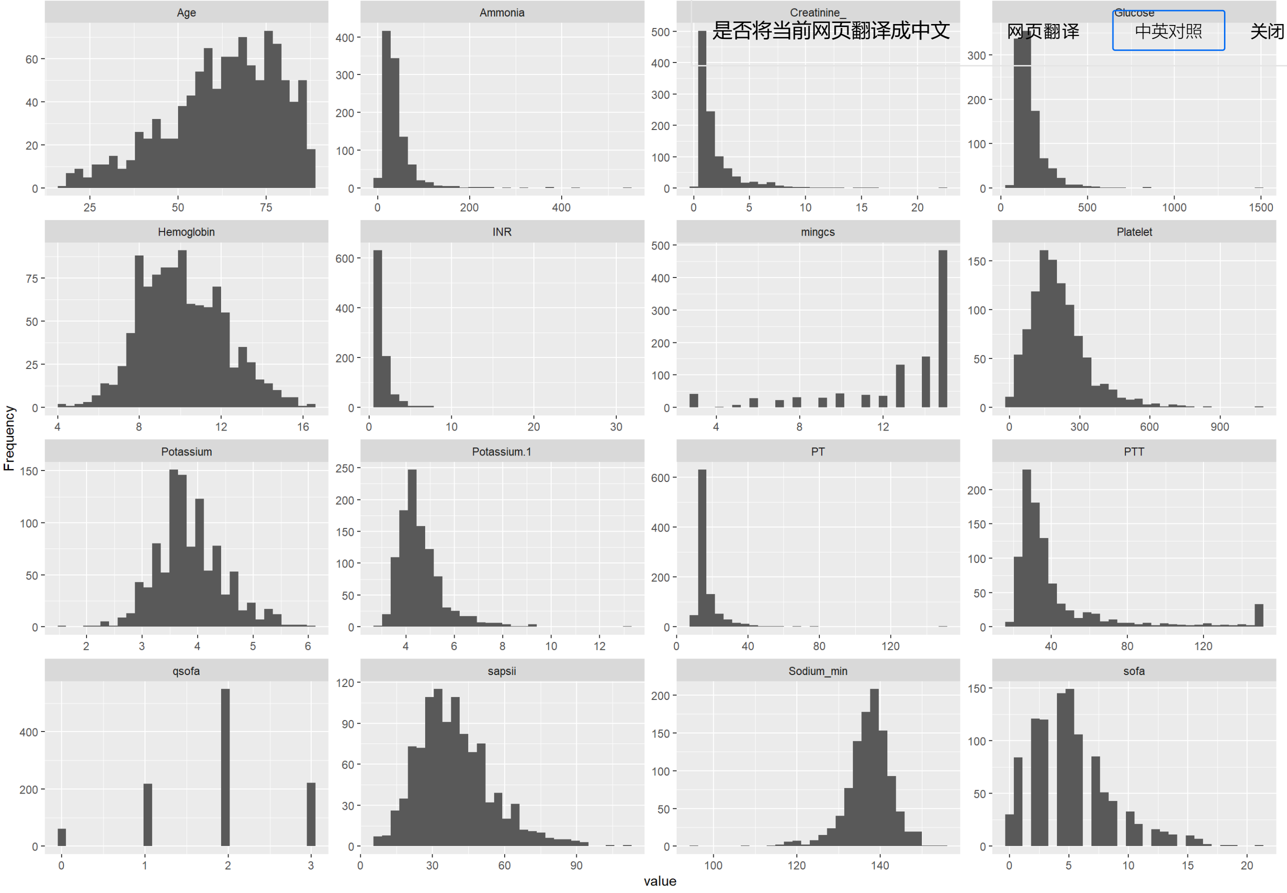

Page 1

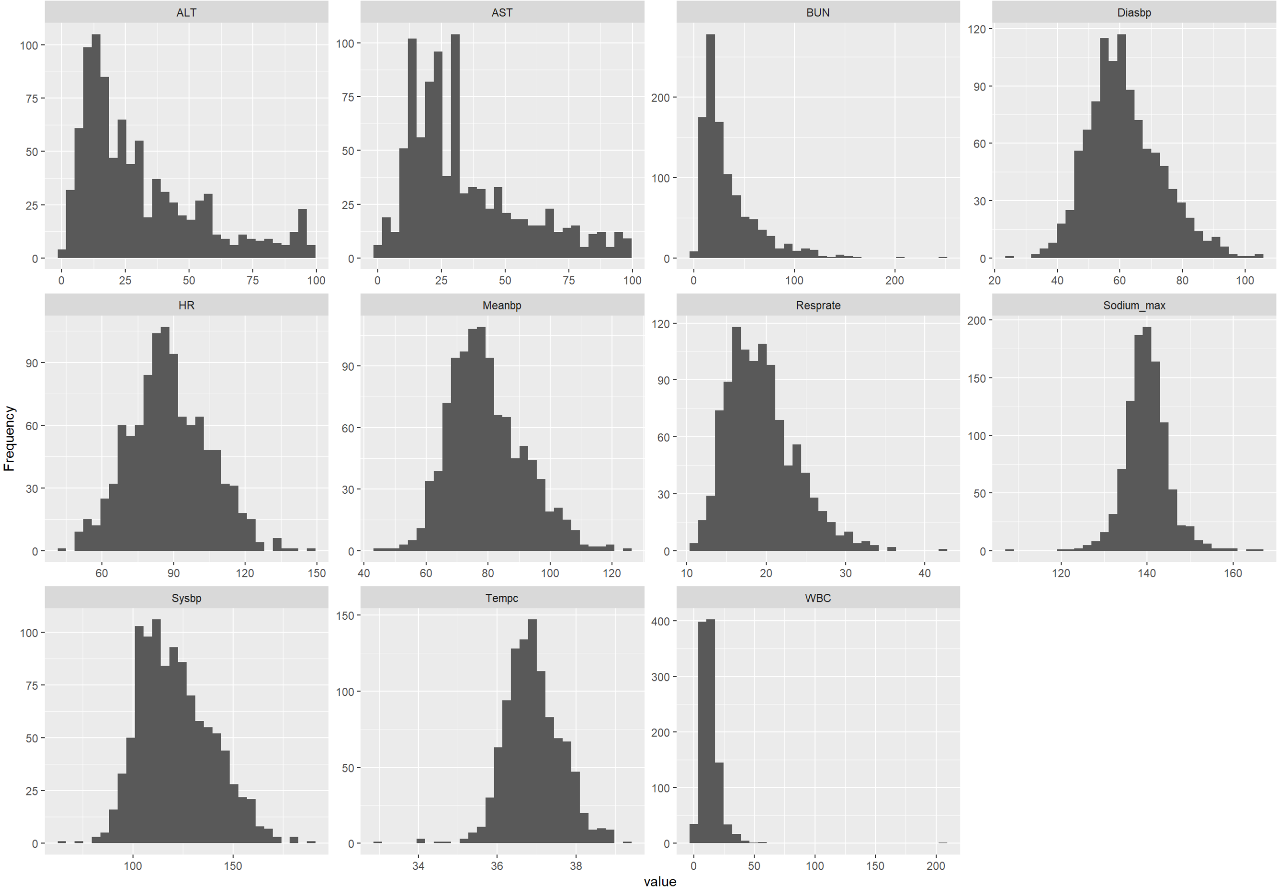

Page 2

Bar Chart (by frequency)

是否将当前网页翻译成中文 网页翻译 中英对照 关闭

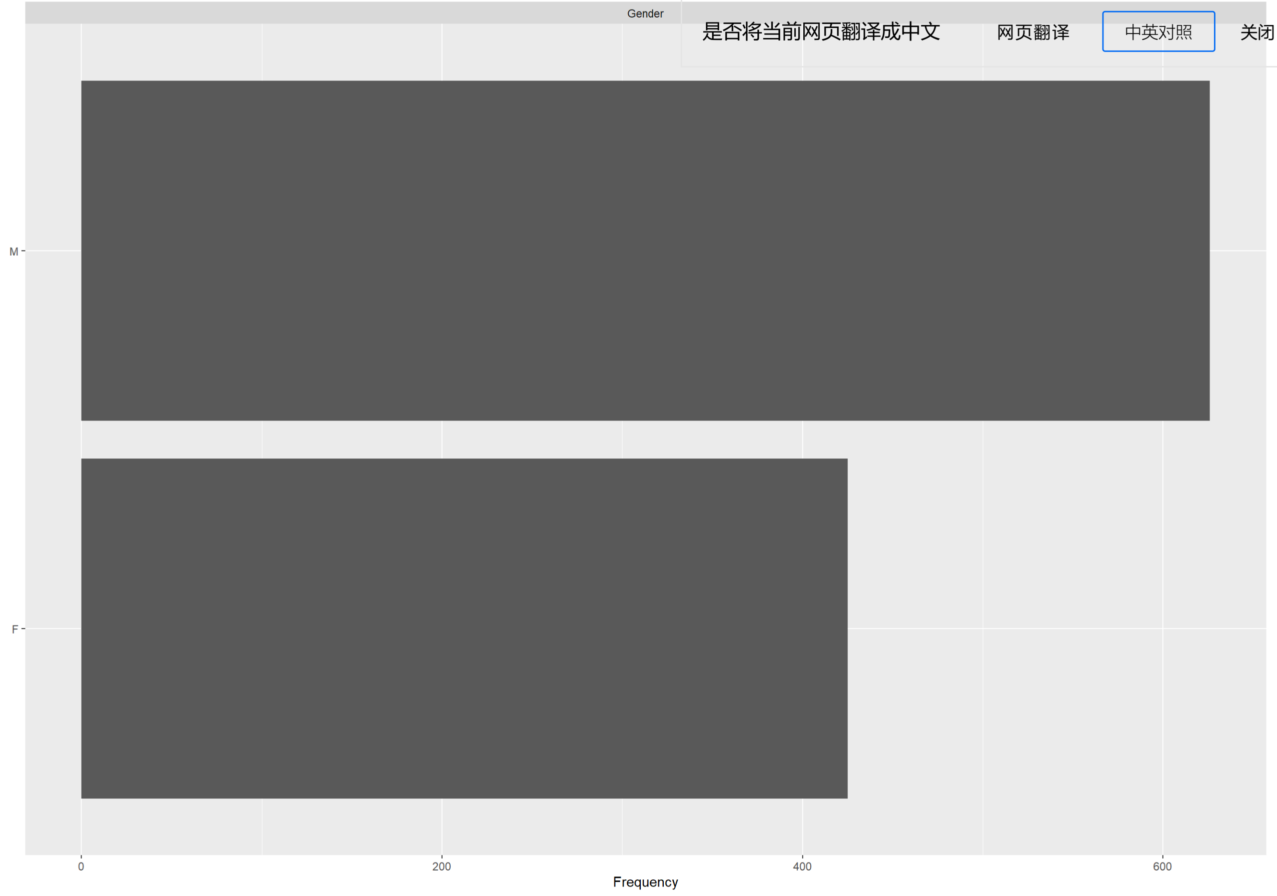

QQ Plot

是否将当前网页翻译成中文 网页翻译 中英对照 关闭

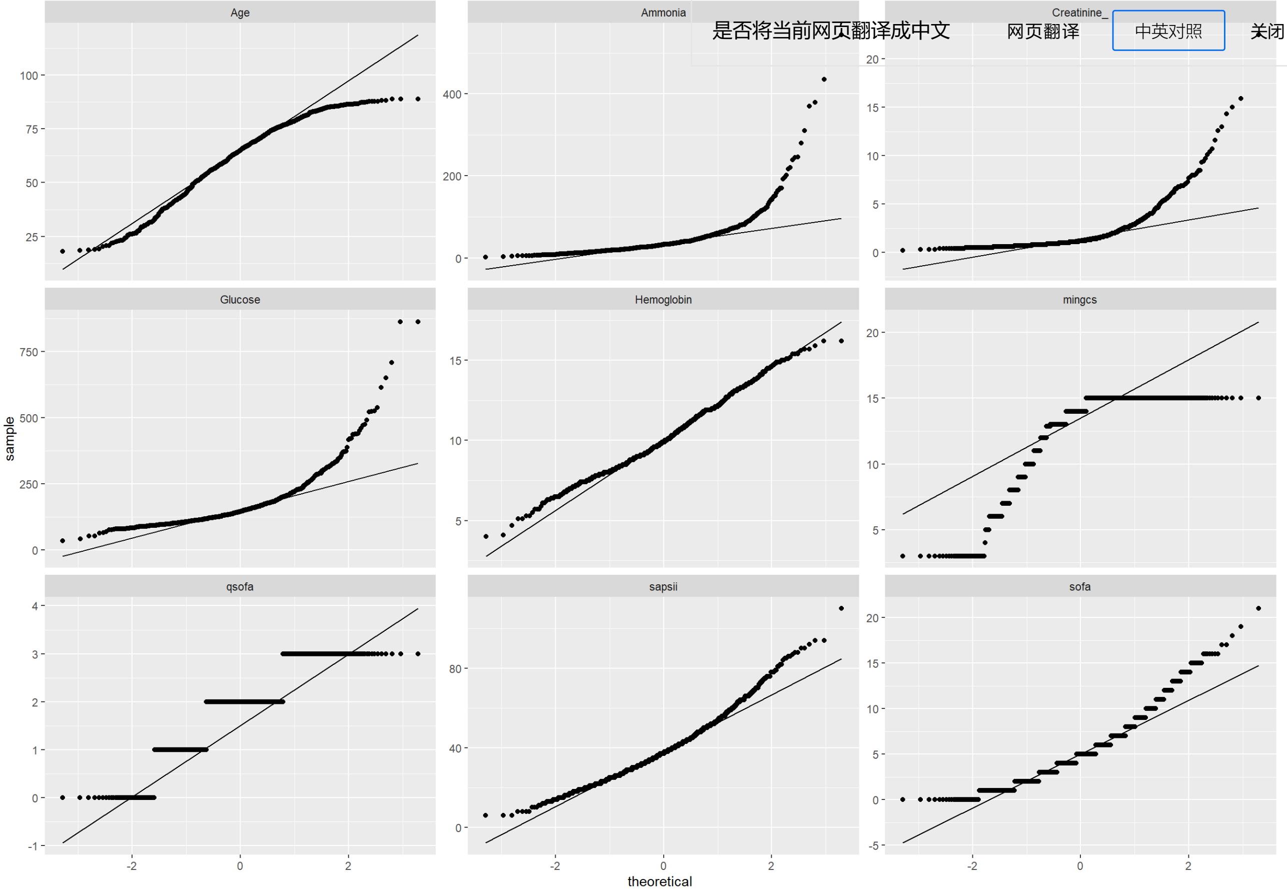

Page 1

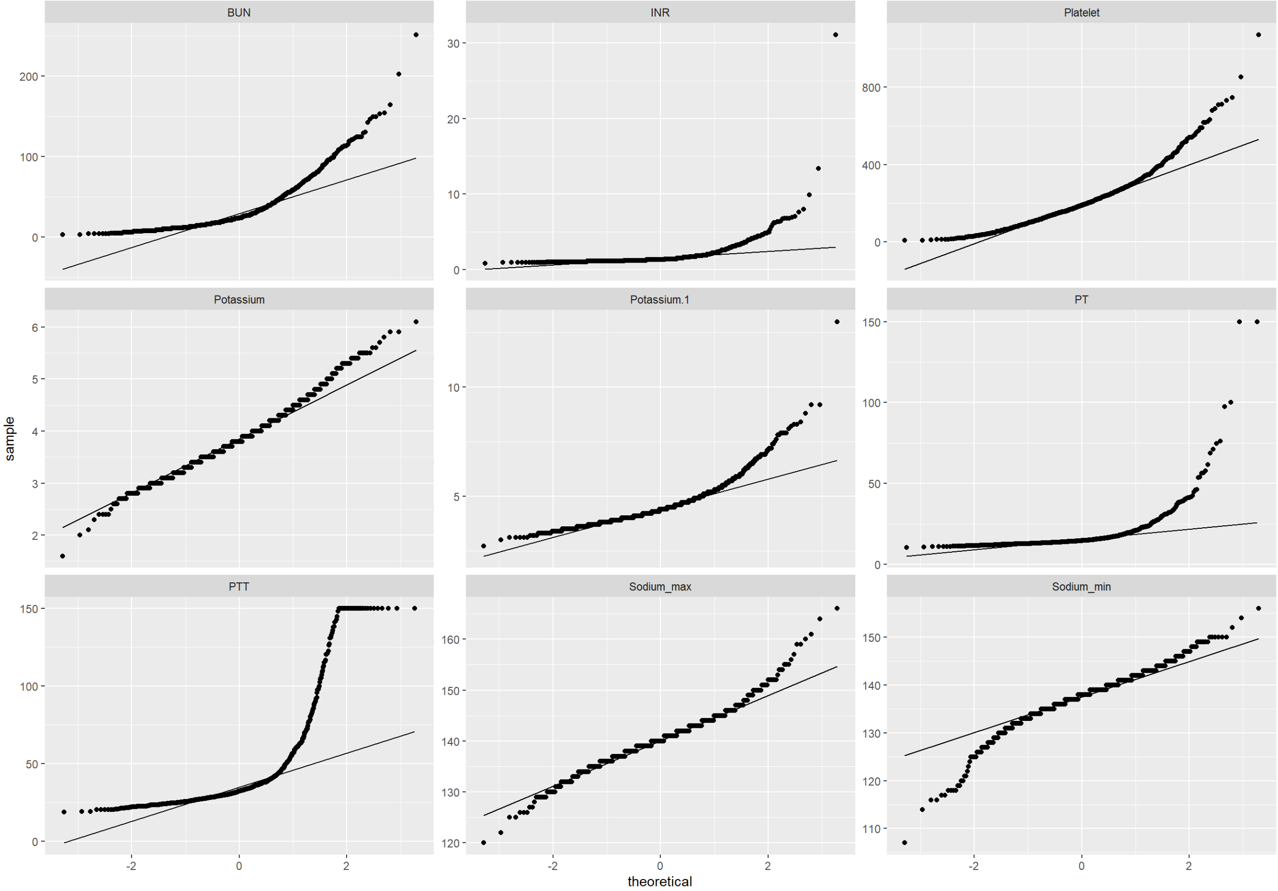

Page 2

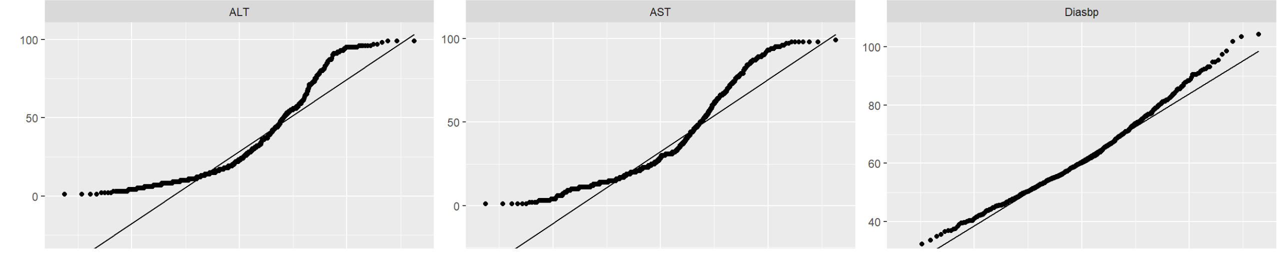

是否将当前网页翻译成中文 网页翻译 中英对照 关闭

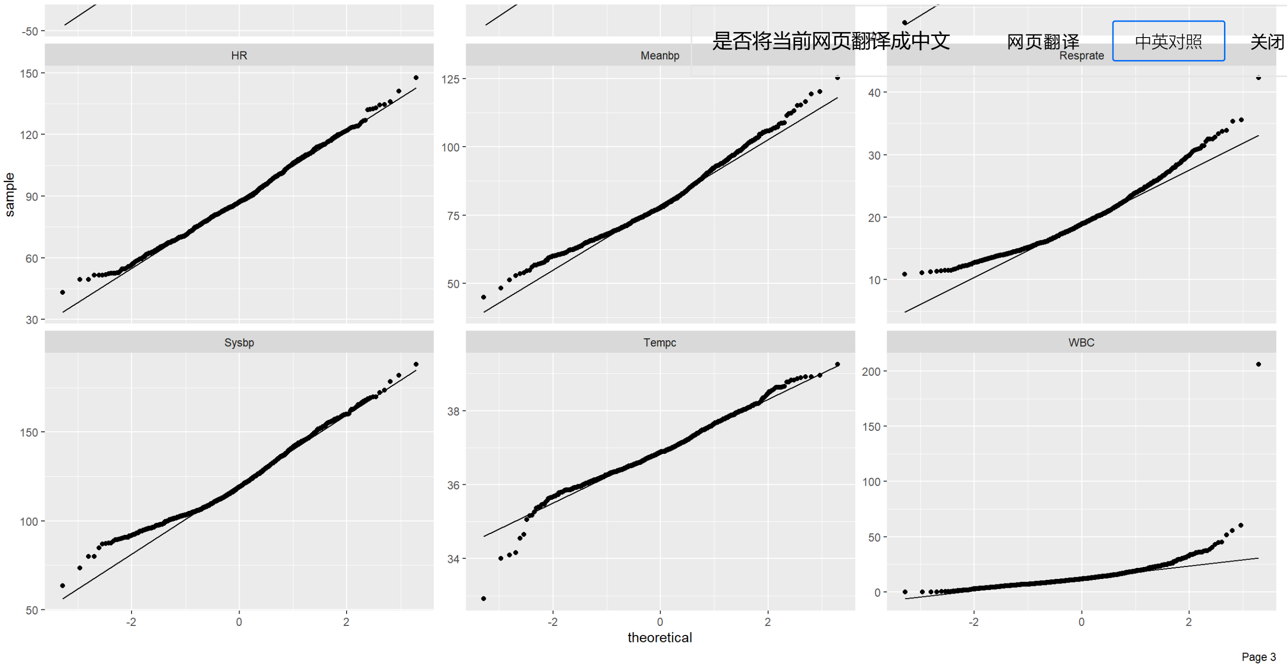

Correlation Analysis

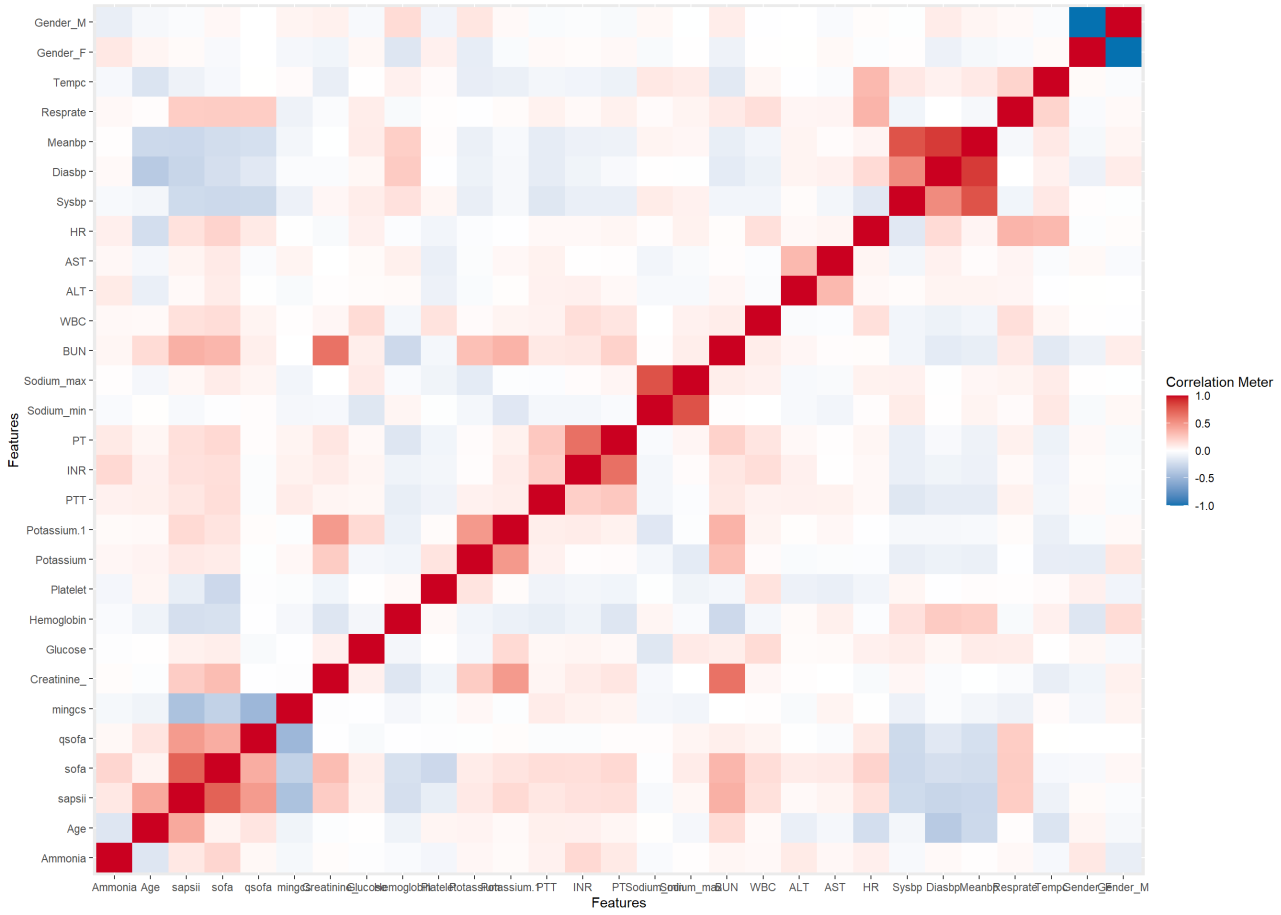

Principal Component Analysis

是否将当前网页翻译成中文 网页翻译 中英对照 关闭

是否将当前网页翻译成中文 网页翻译 中英对照 关闭

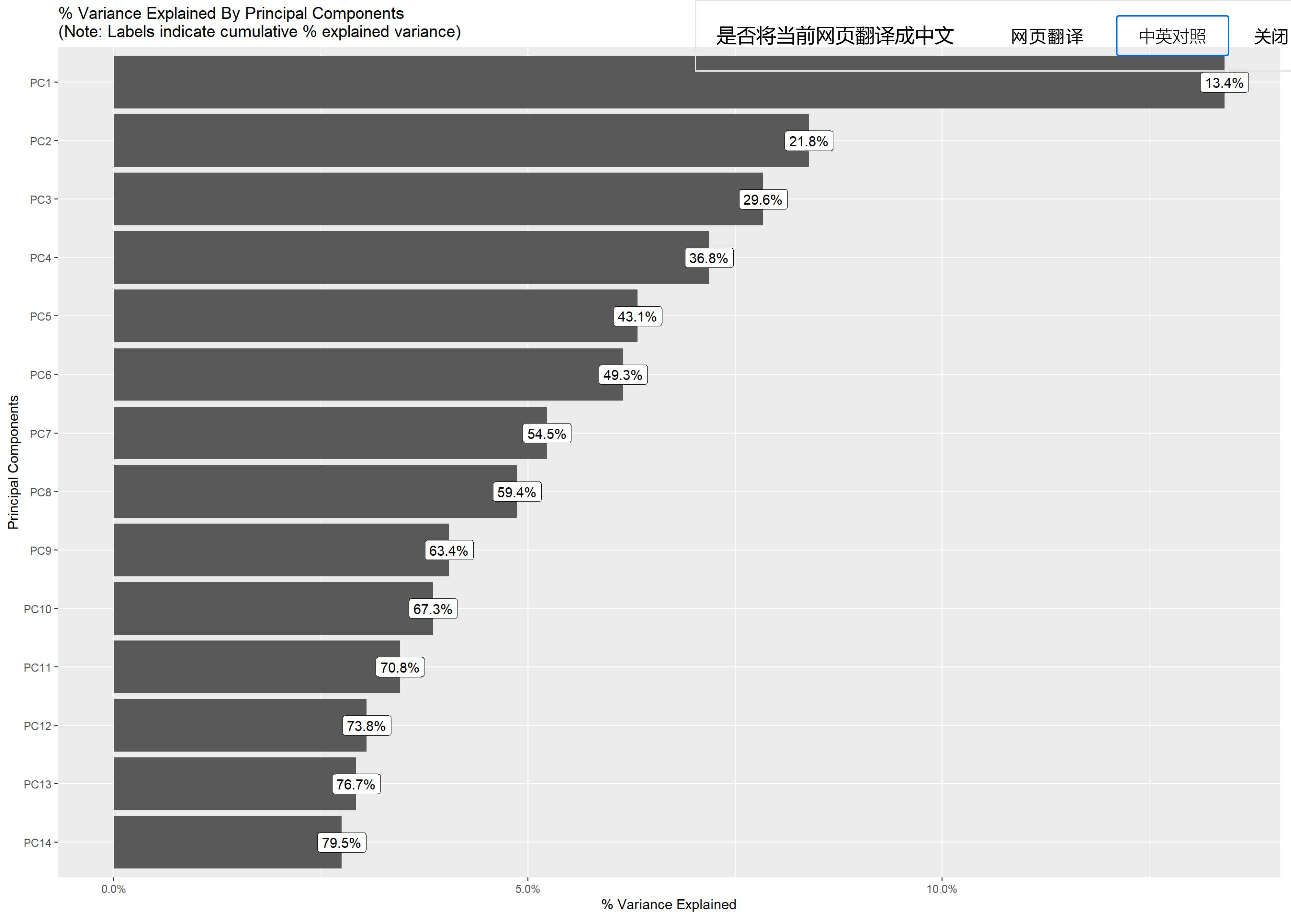

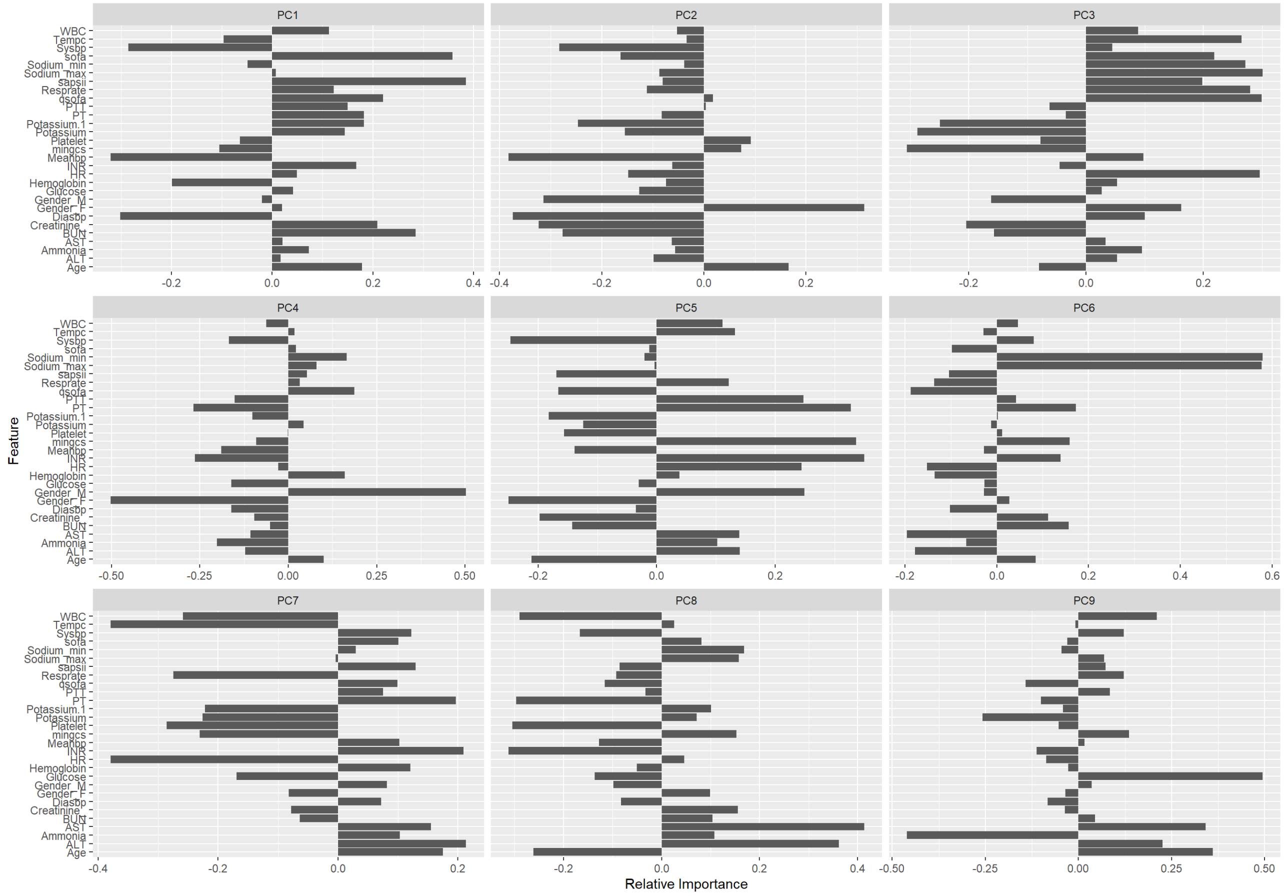

Page 1

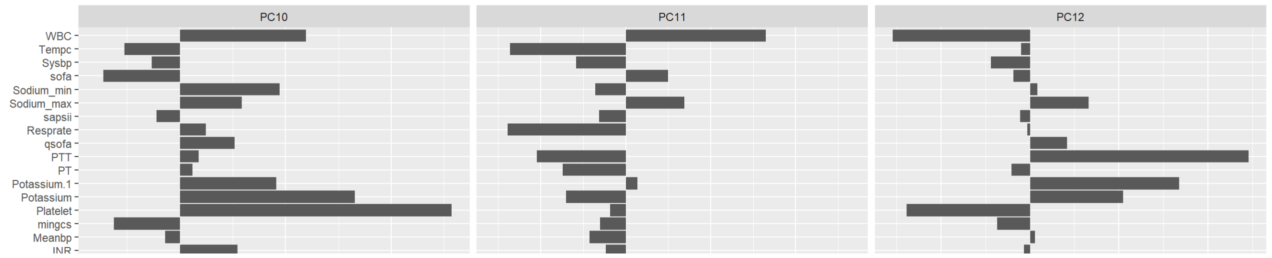

是否将当前网页翻译成中文 网页翻译 中英对照 关闭

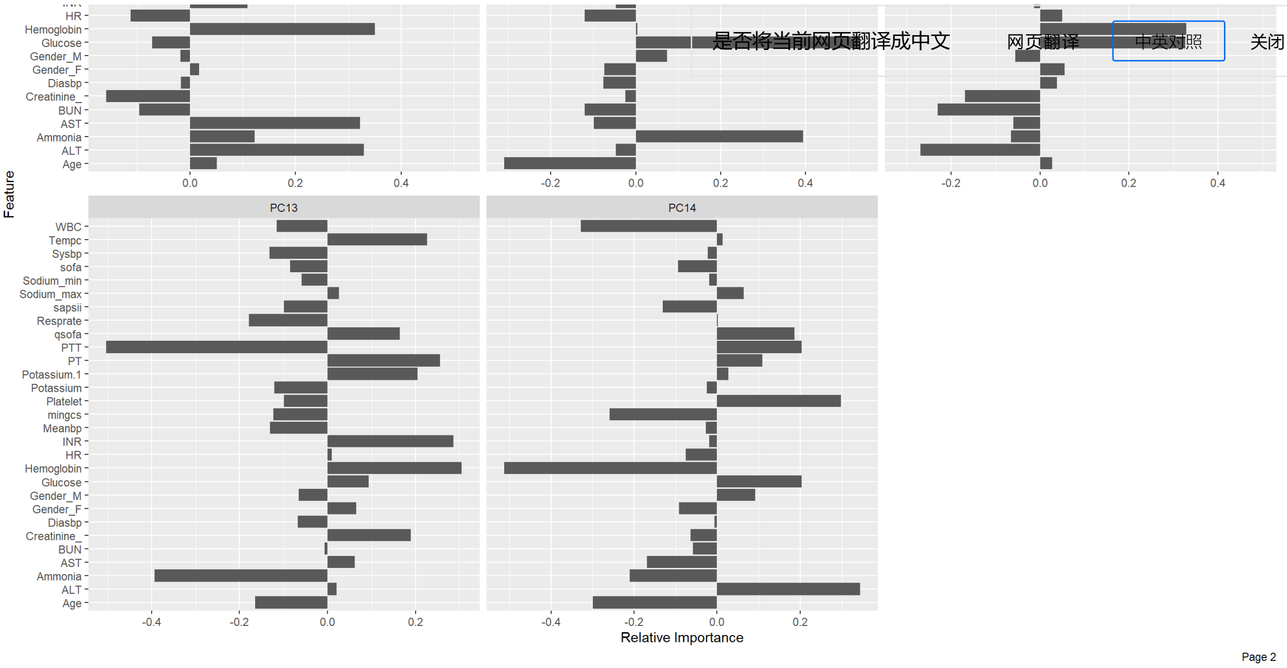

Supplement: Supplementary file 4 [file Data_Sheet_1.PDF]
